# Supplementary material for: Biallelic and gene-wide genomic substitution for endogenous intron and retroelement mutagenesis in human cells
Source: Nat Commun. 2022 Jul 21;13:4219. doi: 10.1038/s41467-022-31982-1 (PMC9304424; doi:10.1038/s41467-022-31982-1)
Supplement: Supplementary file 1 — Supplementary Information [file 41467_2022_31982_MOESM1_ESM.pdf]

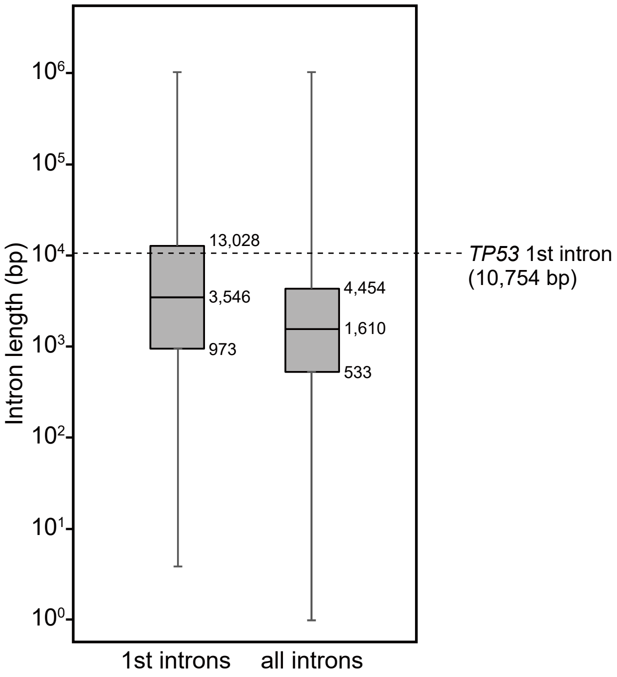

Supplementary Figure 1 | Box plots represent the distribution of base pair length of the 23,702 first introns and the 201,811 all introns within human genes.

The middle line, the box, and the whiskers indicate the median, the 25th to 75th percentiles, and the interquartile range, respectively. The broken line represents the length of the first intron of *TP53*.

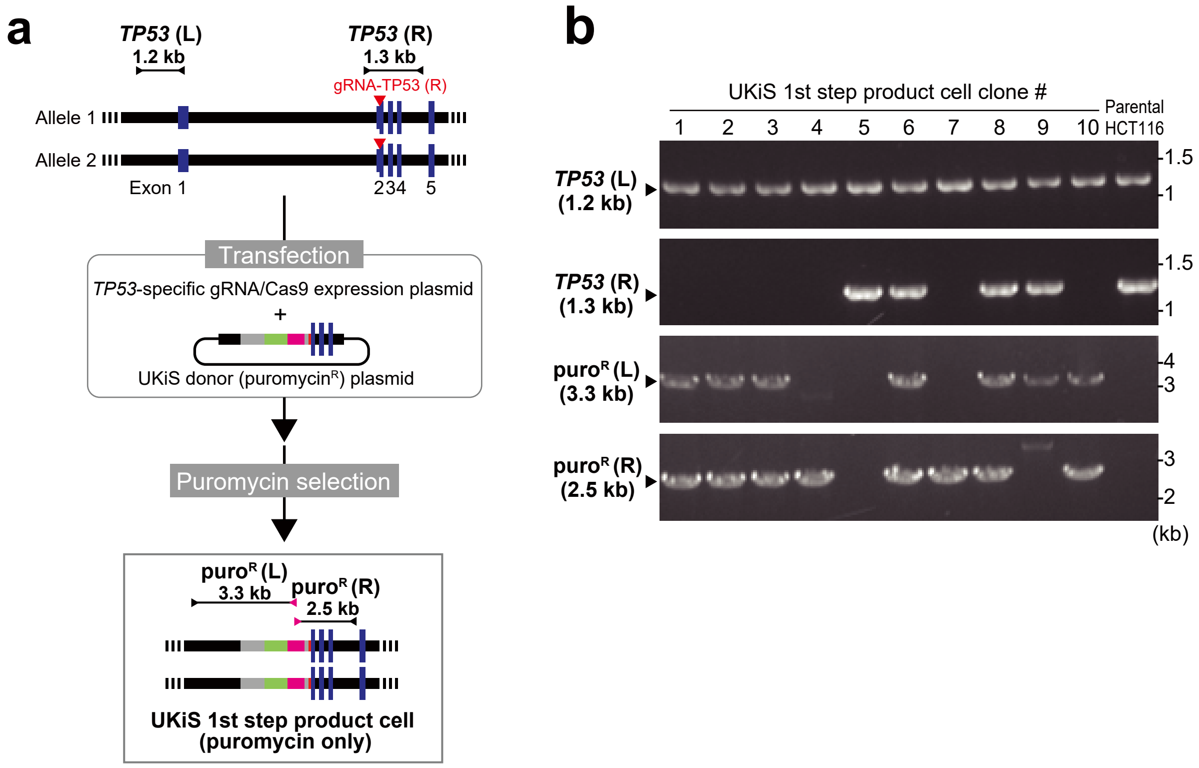

Supplementary Figure 2 | Replacement of the first intron of *TP53* with only one kind of UKiS donor.

a. Schematic of the UKiS first step for replacement of the *TP53* first intron by using only the puromycin UKiS donor. Horizontal lines flanked by two arrowheads represent the target regions for the junction genotyping PCR.

b. Representative gel image of the junction genotyping PCR to confirm deletion of the *TP53* first intron and insertion of the UKiS marker. Of the 10 clones obtained after puromycin selection, none had successful recombination at both alleles. Source data are provided as a Source Data file.

a

Off-target candidate	Sequence	Indel	Mismatch	Chr position
#1	CTCAGGGTGGGCTGGACGCTGGG	0	3	Chr16:89621342-89621364
#2	CTCAGA-AGGGCTCCACGCTTGG	1	2	Chr2:191021507-191021528

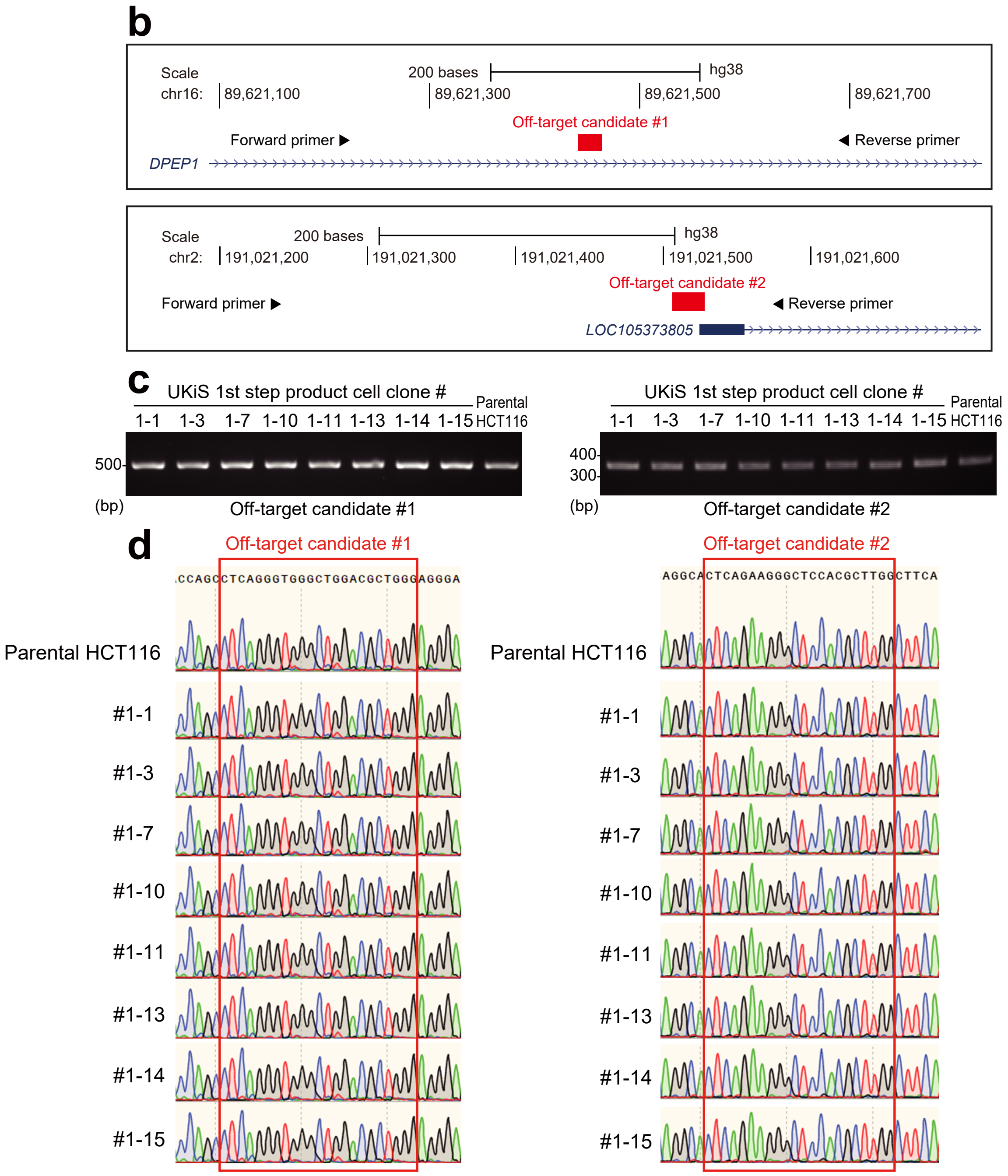

Supplementary Figure 3 | Assessment of off-target cleavage by the *TP53*-specific gRNA.

a. The two most likely off-target candidate sites predicted by COSMID (https://crispr.bme.gatech.edu/). Mismatched nucleotides and a missing nucleotide relative to the *TP53*-specific gRNA sequence are indicated in red. The PAM sequence is underlined.

b. The genomic locations of the off-target candidate sites. The primers used for genomic PCR are denoted by arrowheads. Locations are based on the human genome reference sequence hg38.

c. Representative images of genotyping PCR against the off-target sites by using genomic DNA from the indicated cell clones as the template. Genomic DNA was extracted from the positive clones obtained after the first step of UKiS. All clones were confirmed to have undergone successful recombination of the *TP53* intron with the UKiS donors at both alleles (as shown in Fig. 2c). Source data are provided as a Source Data file.

d. Sequencing of PCR amplicons obtained in (c) demonstrated that no indels or any other genomic modifications occurred at either off-target candidate site, indicating that these sites were not mistakenly cleaved by the *TP53*-specific gRNA.

a

Off-target candidate sequence	Indel	Mismatch	Chr position
GGCGC-AGGCGATCGAGTAA<u>GGG</u>	1	2	Chr19: 35545383-35545404

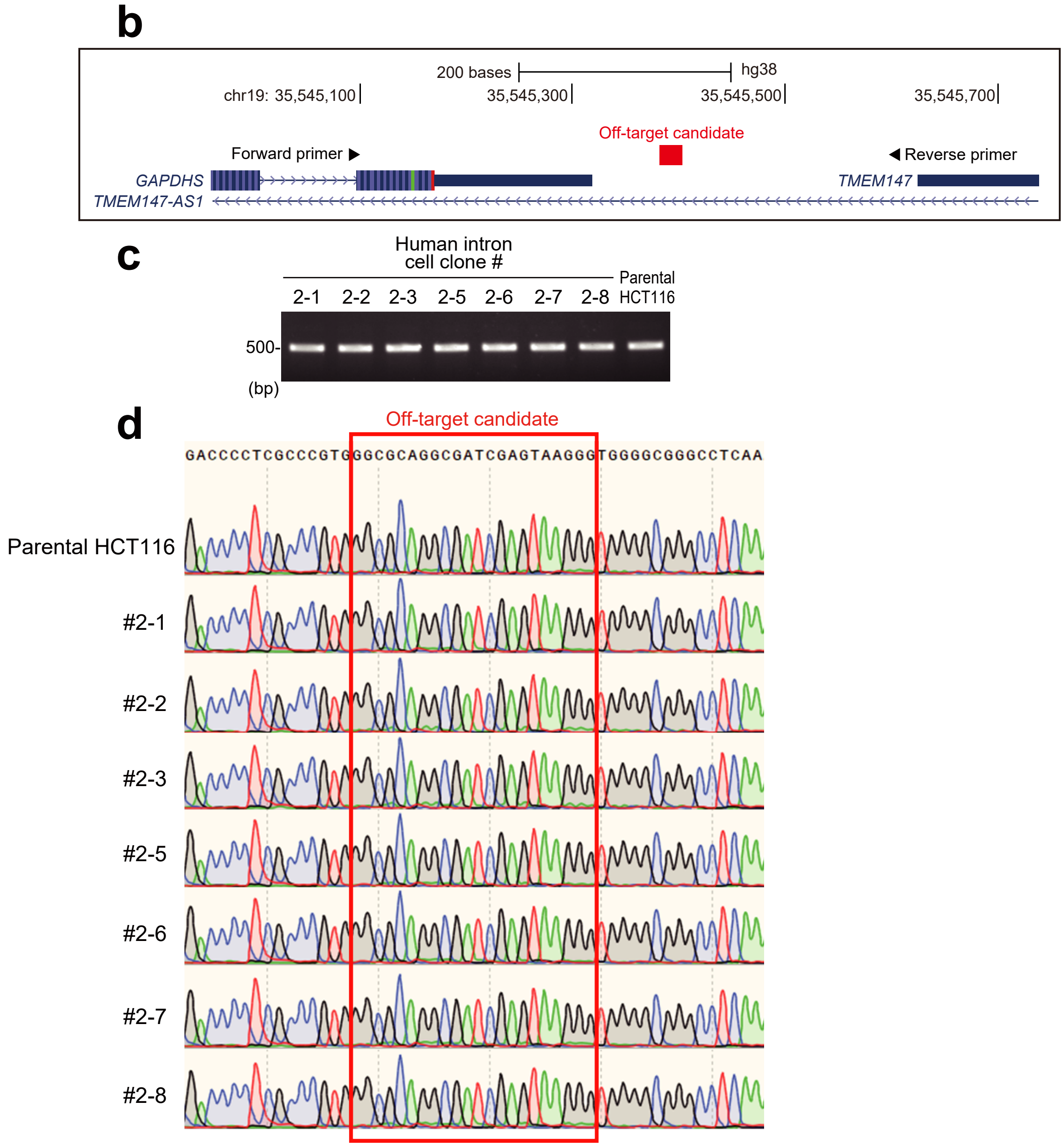

Supplementary Figure 4 | Assessment of off-target cleavage by TL-gRNA.

a. The most likely off-target candidate site predicted by COSMID (https://crispr.bme.gatech.edu/). Mismatched nucleotides and a missing nucleotide relative to the TL-gRNA sequence (5'-GGCGCAACGC-GATCGCGTAA<u>GGG</u>) are indicated in red. The PAM sequence is underlined.

b. The genomic location of the off-target candidate site. The primers used for genomic PCR are denoted by arrowheads.

c. Representative image of genotyping PCR against the off-target site by using genomic DNA from the indicated cell clones as the template. Genomic DNA was extracted from the positive clones obtained after the second step of UKiS with the mutating payload containing the wild-type human intron. As shown in Fig. 3b, these clones were all confirmed to have undergone successful recombination of the UKiS donor allele with the human intron. Source data are provided as a Source Data file.

d. Sequencing of PCR amplicons obtained in (c) demonstrated no indels or any other genomic modifications at the off-target candidate site, indicating that this site was not mistakenly cleaved by the TL-gRNA.

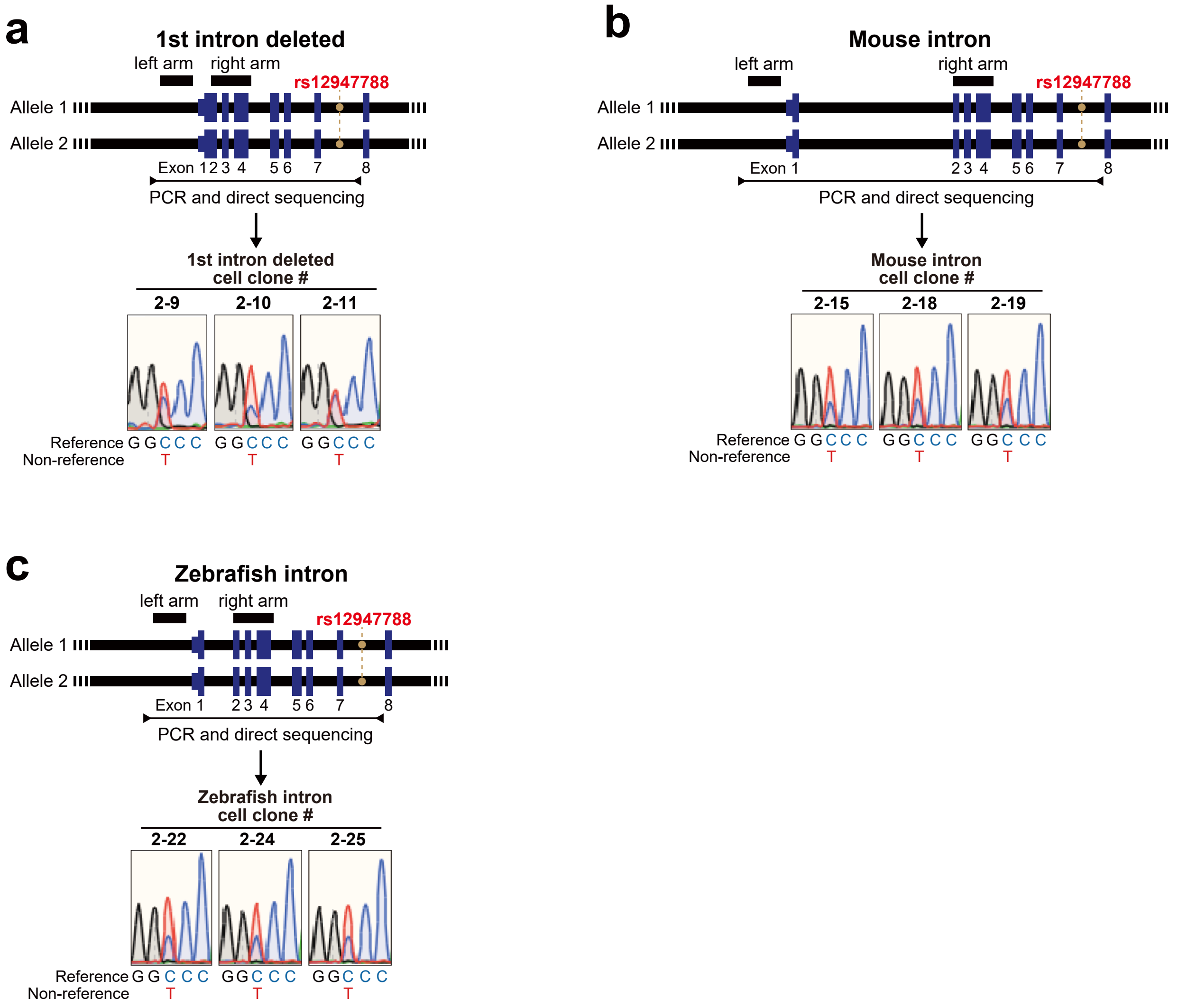

Supplementary Figure 5 | Validation of biallelic replacement in the second step of UKiS to generate three different kinds of *TP53* first intron mutant clones.

For the (a) intron-deleted clones, (b) mouse intron clones, and (c) zebrafish intron clones, graphical representation of the human *TP53* locus is shown on the top: black boxes represent the positions of the homology arms used in our UKiS mutagenesis to *TP53*, horizontal lines flanked by two arrowheads represent the target regions for PCR, and the orange filled circles denote the positions of the heterozygous SNP site, rs12947788. Direct sequencing of the PCR genotyping amplicons indicated double peaks only at rs12947788 on the resultant sequencing chromatograms for all three clones for each kind of mutant.

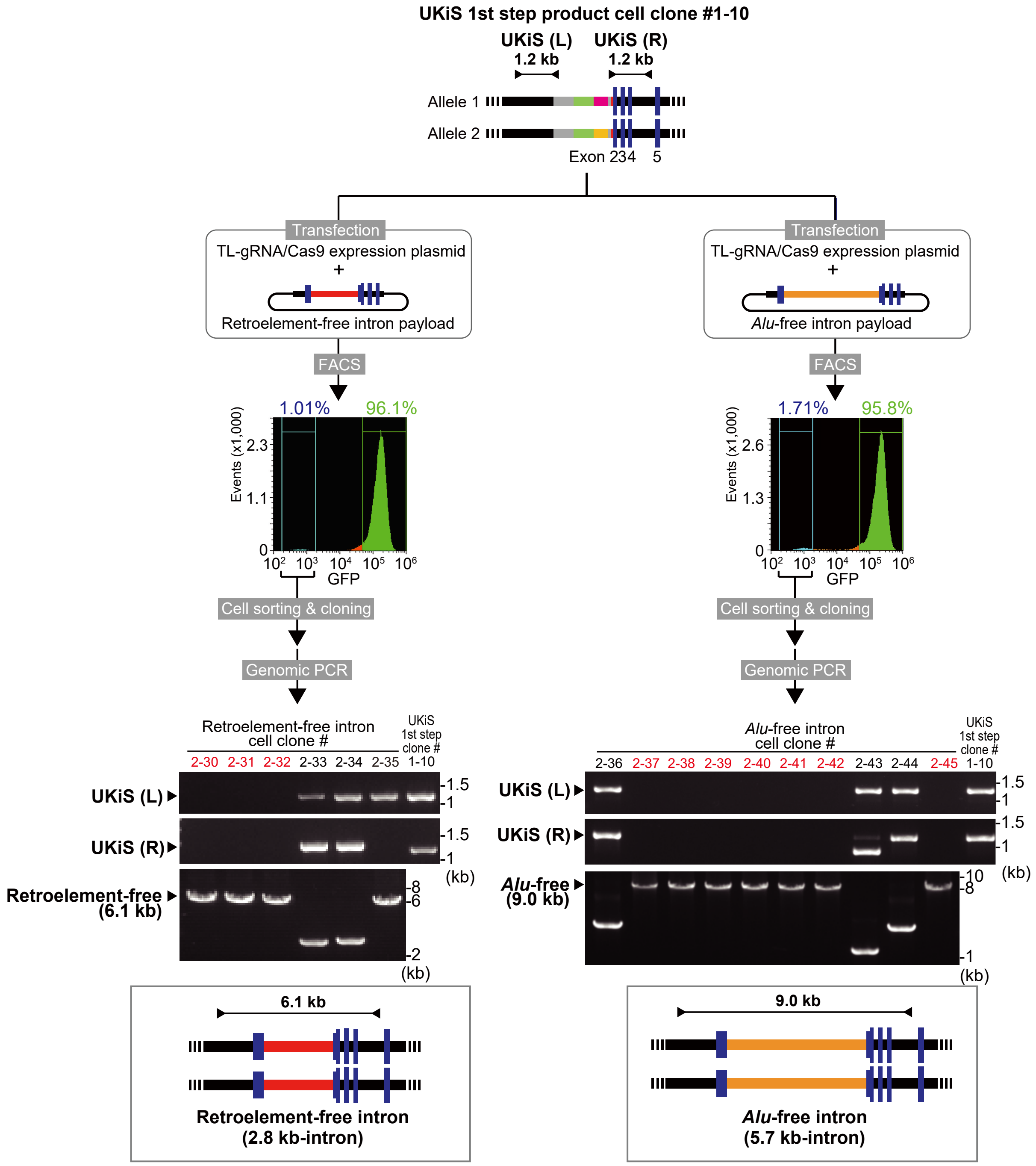

Supplementary Figure 6 | The second step of UKiS: replacement of both UKiS donor alleles in the first intron of *TP53* with either a retroelement- or *Alu*-free intron.
Replacement of both UKiS donor alleles in clone #1-10 with one of two synthetic introns from which all retroelement (left) and *Alu* (right) sequences had been removed. First, the mutating payload plasmid containing the retroelement- or *Alu*-free intron and the TL-gRNA/Cas9 expression plasmid were transfected into clone #1-10. Thereafter, GFP-negative cells were collected by FACS and cloned. Biallelic substitution of UKiS markers with the mutating payload plasmid was confirmed by junction genotyping PCR that targeted the regions represented by horizontal lines flanked by two arrowheads in the schematic diagrams of the *TP53* locus after successful replacement, with the expected length of PCR genotyping amplicons indicated. In the agarose gel images, lane numbers of clones that underwent successful recombination in both alleles are in red. Source data are provided as a Source Data file.

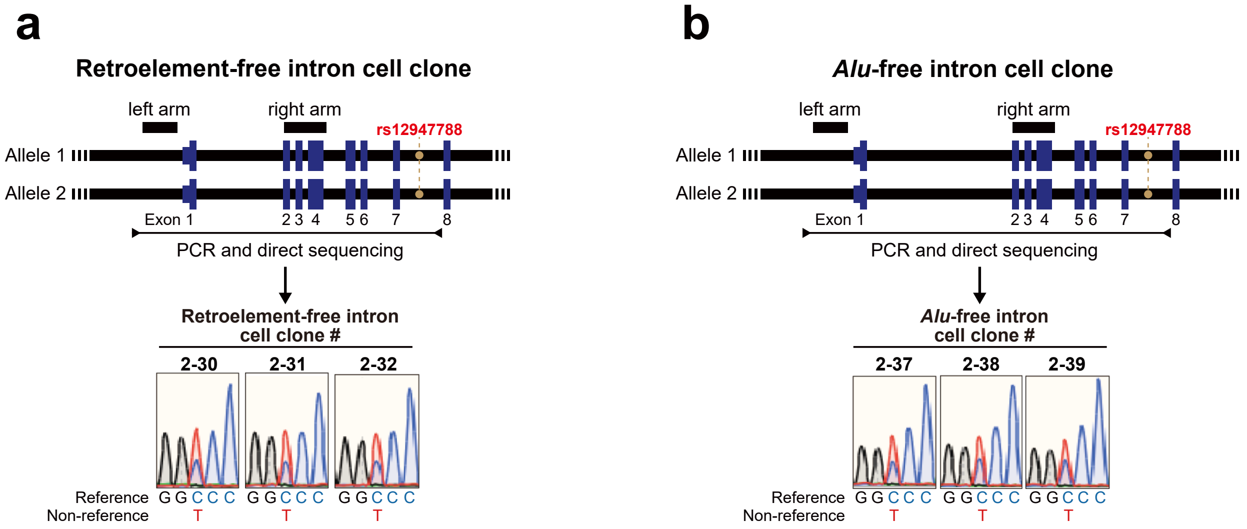

Supplementary Figure 7 | Validation of biallelic replacement in the second step of UKiS for making the retroelement- and *Alu*-free intron clones.

For the (a) retroelement-free intron clone and (b) *Alu*-free intron clone, graphical representation of the human *TP53* locus is shown on the top: black boxes represent the positions of homology arms used in our UKiS mutagenesis to *TP53*, horizontal lines flanked by two arrowheads represent the target regions for the PCR. The orange filled circles denote the positions of the heterozygous SNP site, rs12947788. Direct sequencing of the PCR genotyping amplicons indicated double peaks only at rs12947788 on the resultant sequencing chromatograms for all three clones for both mutants.

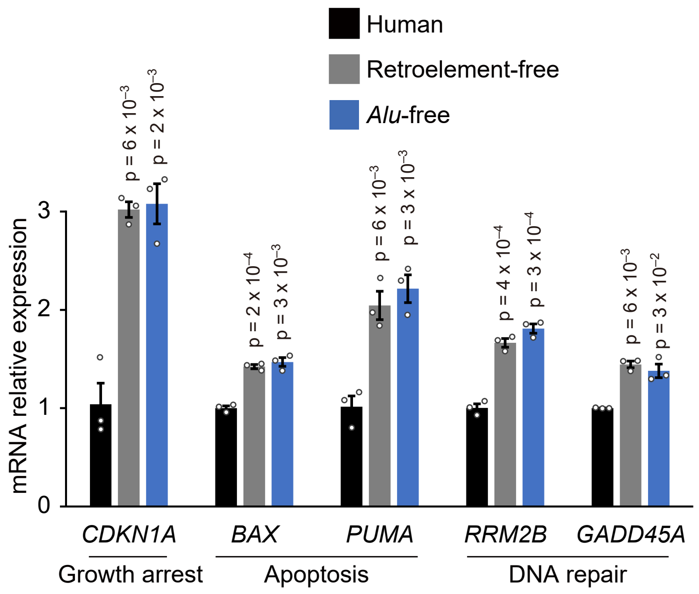

Supplementary Figure 8 | Evaluating the influence of the retroelements and *Alu* elements in the first intron of *TP53* on the expression of *TP53* downstream genes.

Real-time RT-PCR for transcriptional expression of five genes that are regulated by *TP53* in the HCT116 cell clones having the *TP53* first intron that are of full-length human, retroelement-free and *Alu*-free. Three different clones of each intron type that were tested in Fig. 6b were used here. As *TP53*-regulating genes, cyclin dependent kinase inhibitor 1A (*CDKN1A* or *p21*), BCL2 associated X (*BAX*), p53 upregulated modulator of apoptosis (*PUMA*), ribonucleotide reductase regulatory TP53 inducible subunit M2B (*RRM2B* or *p53R2*), and growth arrest and DNA damage inducible alpha (*GADD45A*) were examined. 18S rRNA was used as an internal control. Reactions were run in duplicate in three independent experiments. Data represent the mean ± SD of experimental results of three independent cell clones, and the p-values (relative to the human intron clones) were calculated based on three independent experiments using three independent cell clones by a two-tailed Student's t-test. Source data are provided as a Source Data file.

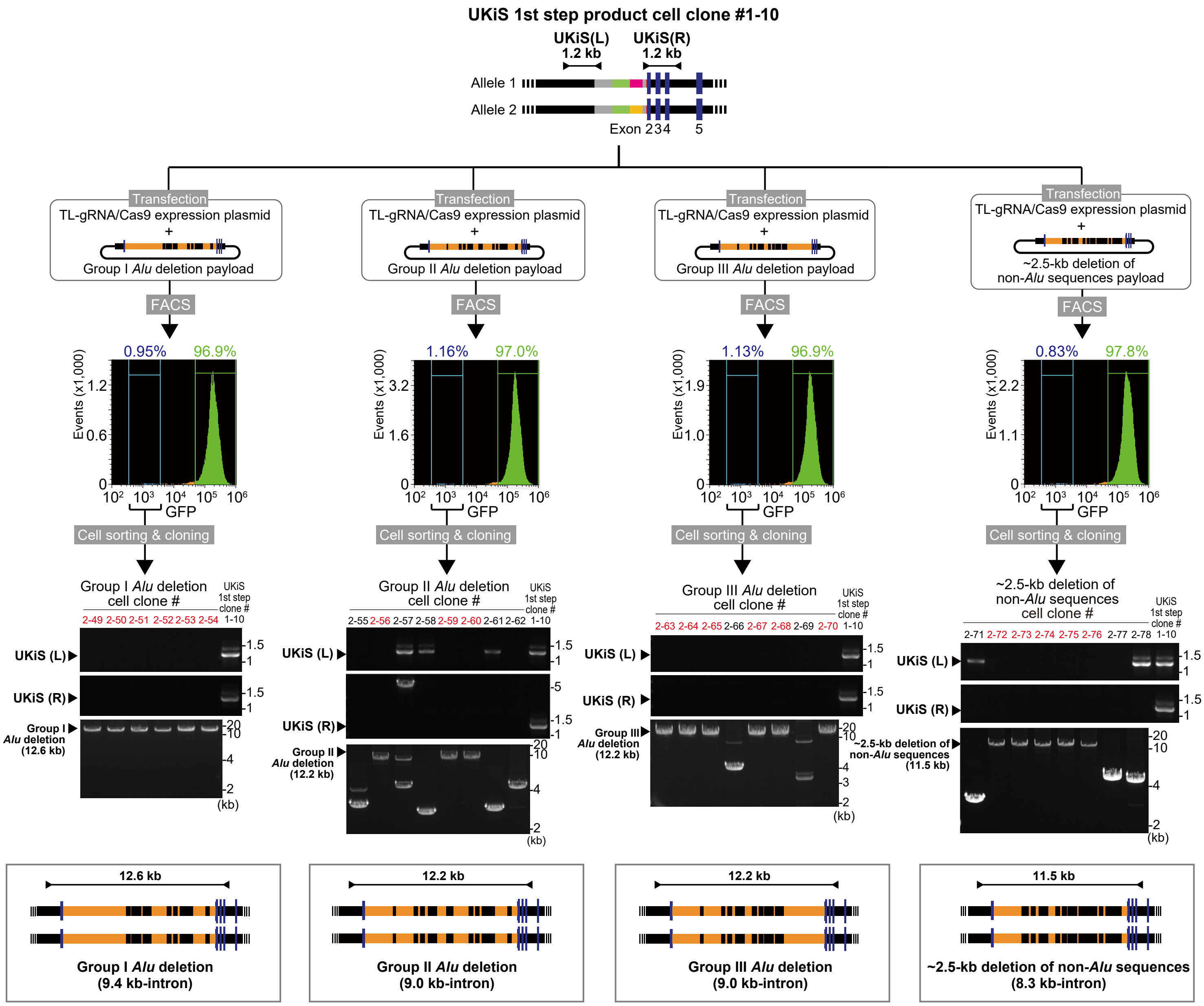

Supplementary Figure 9 | Replacement of both UKiS donor alleles with the first intron of *TP53* that contains only a subset of *Alu* sequences or that lacks ~2.5kb of non-*Alu* sequences.

Replacement of both UKiS donor alleles with synthetic introns in which some of the *Alu* sequences or non-*Alu* sequences have been removed. First, the mutating payload plasmids containing the partial deletion of *Alu* sequences or deletion of non-*Alu* sequences from the first intron of *TP53* and the TL-gRNA/Cas9 expression plasmid were transfected into clone #1-10. Thereafter, GFP-negative cells were collected by FACS and cloned. Biallelic substitution of UKiS markers with the mutating payload plasmid was confirmed by junction genotyping PCR, which targeted the regions represented by horizontal lines flanked by two arrowheads in the schematic diagrams of the *TP53* locus after successful replacement. In the agarose gel images, lane numbers of clones that underwent successful recombination in both alleles are in red. Source data are provided as a Source Data file.

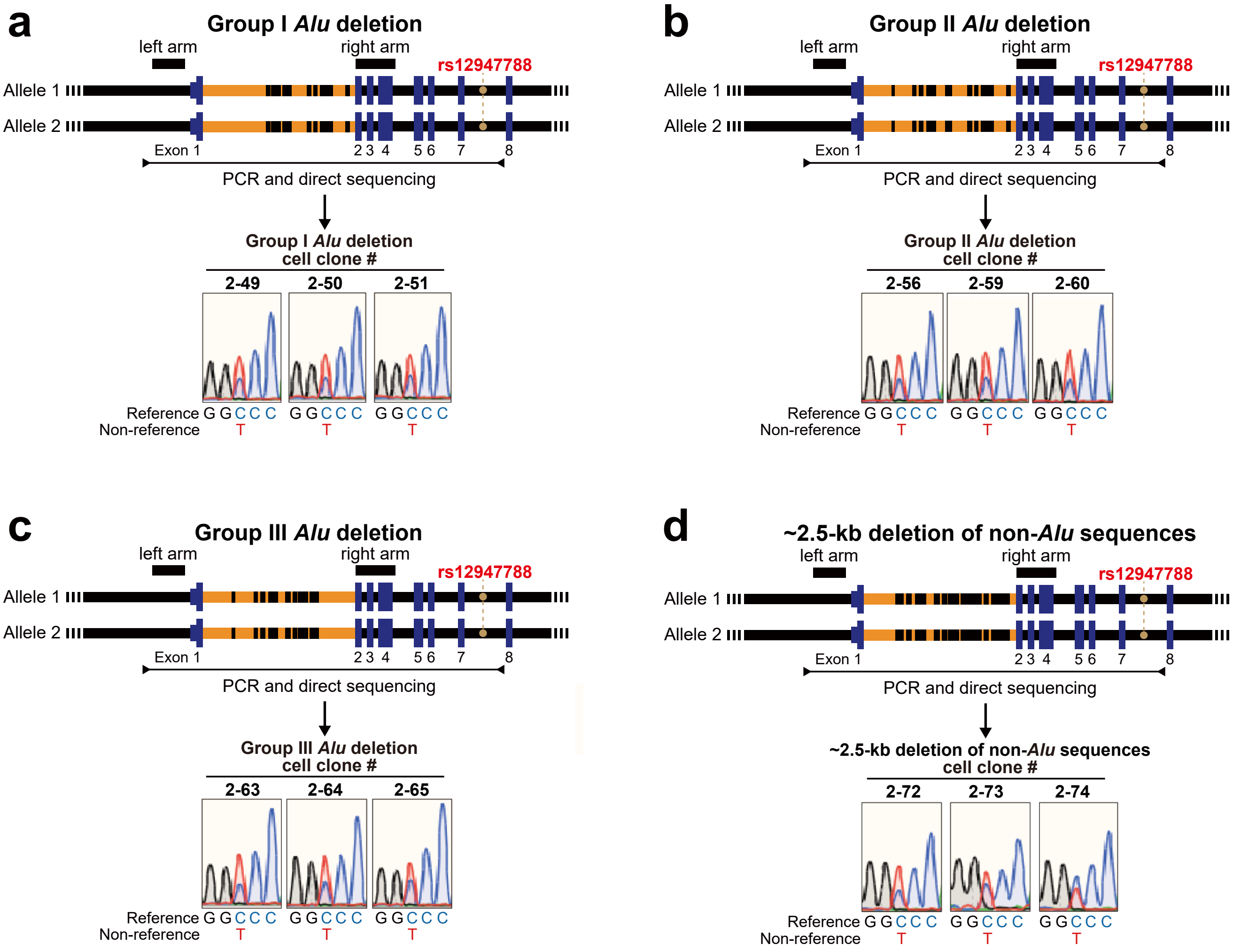

Supplementary Figure 10 | Validation of biallelic replacement among clones with the first intron of *TP53* that contains only a subset of *Alu* sequences or that lacks ~2.5kb of non-*Alu* sequences.
For each of the (a) Group I *Alu* deletion, (b) Group II *Alu* deletion, and (c) Group III *Alu* deletion clones and the (d) ~2.5-kb deletion of non-*Alu* sequences clones, graphical representation of the human *TP53* gene locus is shown on the top: black boxes represent the positions of homology arms used in our UKiS mutagenesis to *TP53*, horizontal lines flanked by two arrowheads represent the target regions for the PCR, and the orange filled circles denote the positions of the heterozygous SNP site, rs12947788. Direct sequencing of the PCR genotyping amplicons indicated double peaks only at rs12947788 on the resultant sequencing chromatograms for all three clones of each mutant.

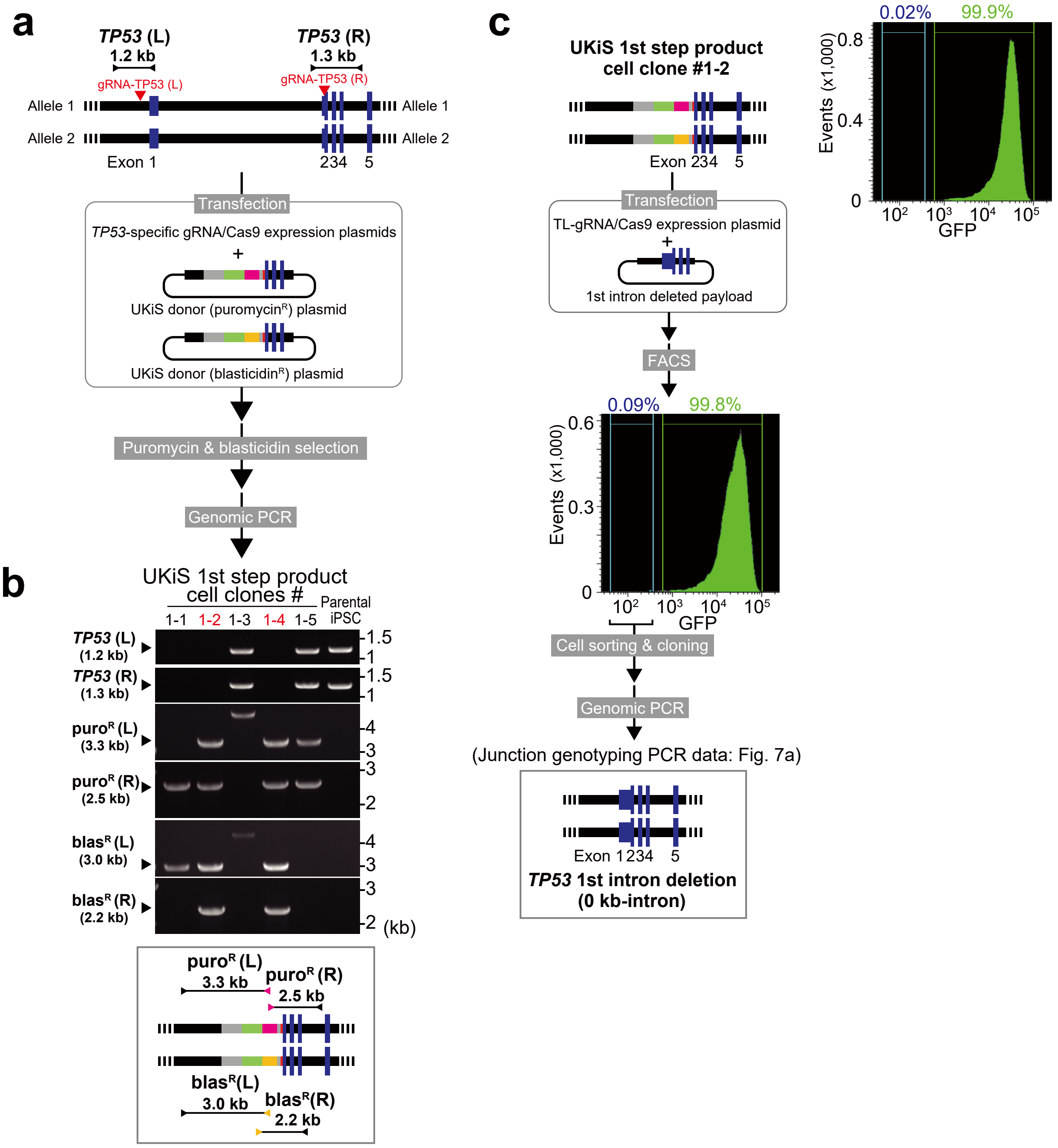

Supplementary Figure 11 | UKiS deletion of the first intron of *TP53* in iPS cells.

a. Schematic diagram of the first step of UKiS for the first intron of *TP53*. Both UKiS donor plasmids and two *TP53*-specific gRNA/Cas9 expression plasmids were transfected into iPS cells, leading to isolation of cell clones that underwent homologous recombination within the *TP53* locus after dual selection with puromycin and blasticidin. Horizontal lines flanked by two arrowheads represent the target regions for the junction genotyping PCR, with the expected length of PCR genotyping amplicons indicated.

b. Representative gel images of the junction genotyping PCR to confirm deletion of the first intron of *TP53* and insertion of UKiS markers. Of the five selected clones, two (shown in red) had successful replacement of the intron with the UKiS donor in both alleles. Source data are provided as a Source Data file.

c. Schematic diagram of the second step of UKiS for replacement of both UKiS donor alleles with the *TP53* sequence with the first intron deleted. The payload plasmid with the first intron deleted and the TL-gRNA/-Cas9 expression plasmid were transfected into the iPS cell clone #1-2, which was obtained in (b) and mostly GFP-positive as shown by FACS analysis. Thereafter, GFP-negative cells were collected by FACS and cloned. Biallelic substitution of UKiS markers with the mutating payload plasmid was confirmed by junction genotyping PCR. A representative image of the resulting agarose gel electrophoresis of junction PCR amplicons is shown in Fig. 7a.

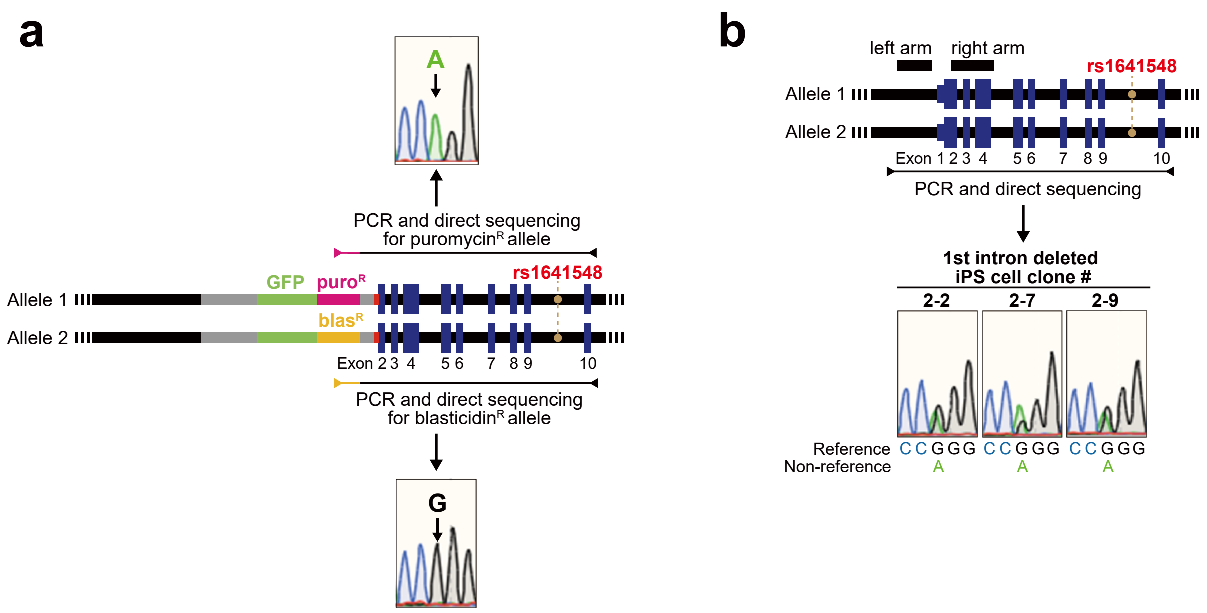

Supplementary Figure 12 | Validation of biallelic replacement in the second step of UKiS to delete the first intron of *TP53* in iPS cell clones.

a. Genotyping of the iPS cell clone #1-2, which was obtained after the first step of UKiS (Supplementary Fig. 11b) and was used to create the deletion of the first intron of *TP53* in iPS cell clones (Fig. 7a). Allele-specific PCR was performed by using primers for puromycin or blasticidin marker sequences, demonstrating that the puromycin and blasticidin alleles had A and G at rs1641548, respectively. Horizontal lines flanked by two arrowheads represent the target regions for the PCR of each allele.

b. Graphical representation of the human *TP53* locus is shown on the top: black boxes represent the positions of homology arms used in our UKiS mutagenesis to *TP53*, the horizontal line flanked by two arrowheads represents the target region for PCR, and the orange filled circles denote the positions of the heterozygous SNP site, rs1641548. Direct sequencing of the PCR genotyping amplicons indicated double peaks only at rs1641548 on the resultant sequencing chromatograms for all three clones of the mutant.

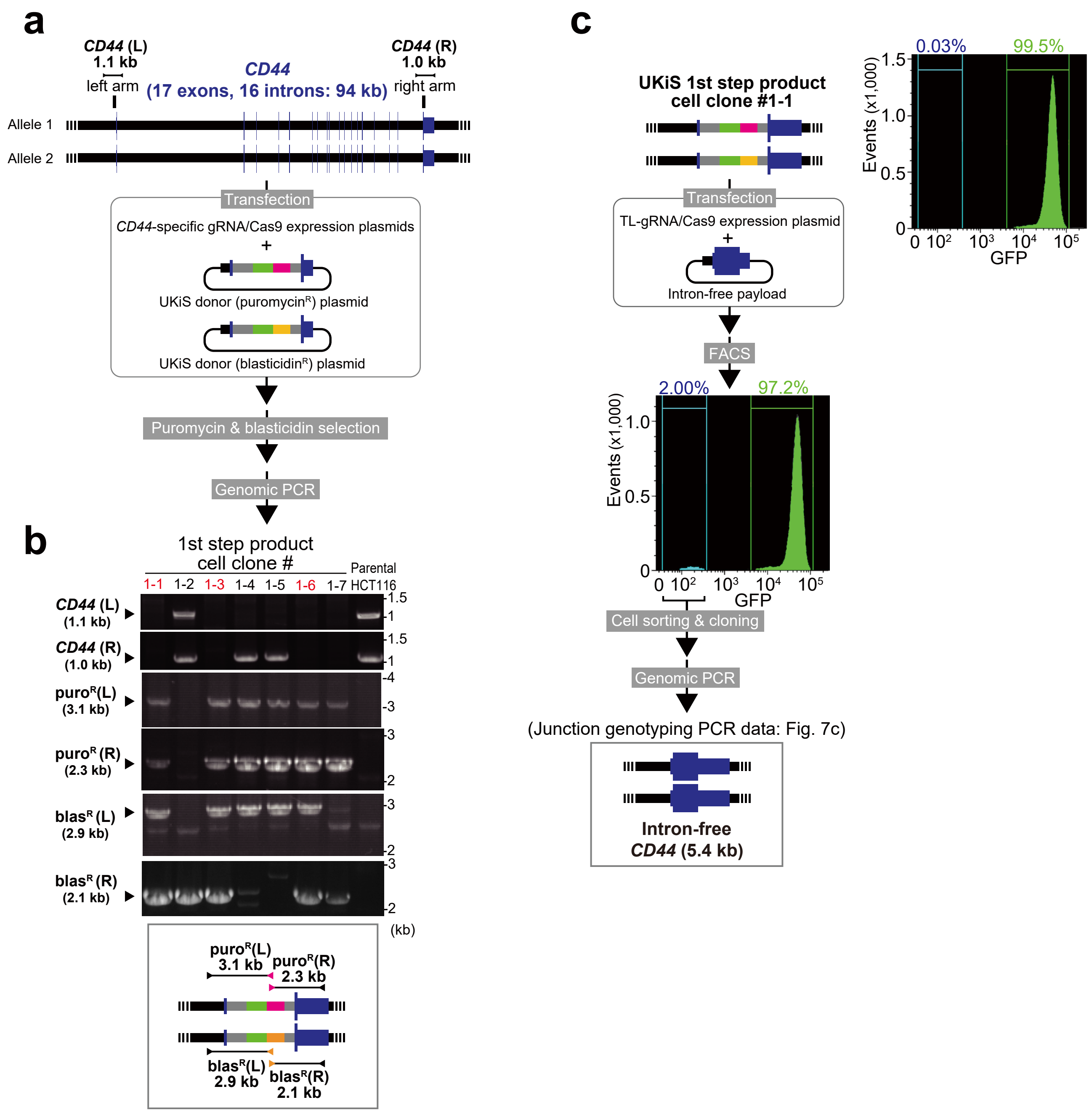

Supplementary Figure 13 | Deletion of all introns from *CD44* in HCT116 cells by UKiS.

a. Schematic diagram of the first step of UKiS used to generate intron-free *CD44* cell clones. Both UKiS donor plasmids and two *CD44*-specific gRNA/Cas9 expression plasmids were transfected into HCT116 cells, leading to isolation of cell clones that underwent homologous recombination within the *CD44* locus after dual selection with puromycin and blasticidin. Horizontal lines flanked by two arrowheads represent the target regions for junction genotyping PCR, with the expected length of PCR genotyping amplicons indicated.

b. Representative gel image of the junction genotyping PCR to confirm deletion of *CD44* and insertion of the UKiS markers. Of the seven selected clones, three (shown in red) had successful replacement of the *CD44* sequence with the UKiS donor in both alleles. Source data are provided as a Source Data file.

c. Schematic diagram of the second step of UKiS during which both UKiS donor alleles were replaced with the intron-free *CD44* sequence. First, the intron-free *CD44* payload plasmid and the TL-gRNA/Cas9 expression plasmid were transfected into clone #1-1, which was obtained in (b) and mostly GFP-positive (99.5%) as shown by FACS analysis. Thereafter, GFP-negative cells were collected by FACS and cloned. Biallelic substitution of UKiS markers with the mutating payload plasmid was confirmed by junction genotyping PCR (Supplementary Fig. 16). A representative image of the resulting agarose gel electrophoresis of junction PCR amplicons is shown in Figure 7c.

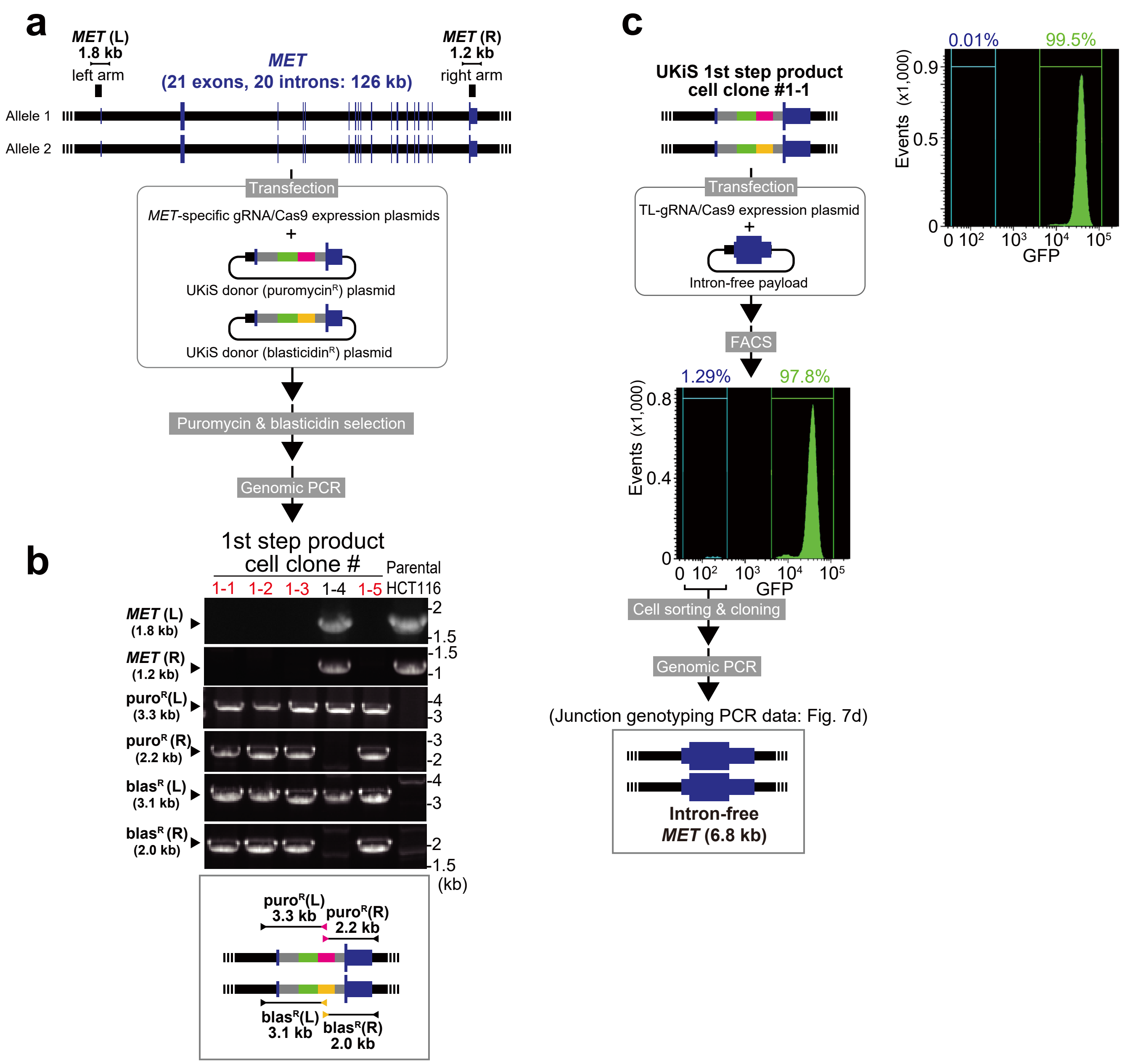

Supplementary Figure 14 | Deletion of all introns from *MET* in HCT116 cells by UKiS.

a. Schematic diagram of the first step of UKiS used to generate intron-free *MET* cell clones. Both UKiS donor plasmids and two *MET*-specific gRNA/Cas9 expression plasmids were transfected into HCT116 cells, leading to isolation of cell clones that underwent homologous recombination within the *MET* locus after dual selection with puromycin and blasticidin. Horizontal lines flanked by two arrowheads represent the target regions for junction genotyping PCR, with the expected length of PCR genotyping amplicons indicated.

b. Representative gel image of the junction genotyping PCR to confirm deletion of *MET* and insertion of the UKiS markers. Of the five selected clones, four (shown in red) had successful replacement of the *MET* sequence with the UKiS donor in both alleles. Source data are provided as a Source Data file.

c. Schematic diagram of the second step of UKiS during which both UKiS donor alleles were replaced with the intron-free *MET* sequence. First, the intron-free *MET* payload plasmid and the TL-gRNA/Cas9 expression plasmid were transfected into clone #1-1, which was obtained in (b) and mostly GFP-positive (99.5%) as shown by FACS analysis. Thereafter, GFP-negative cells were collected by FACS and cloned. Biallelic substitution of UKiS markers with the mutating payload plasmid was confirmed by junction genotyping PCR (Supplementary Fig. 17). A representative image of the resulting agarose gel electrophoresis of junction PCR amplicons is shown in Figure 7d.

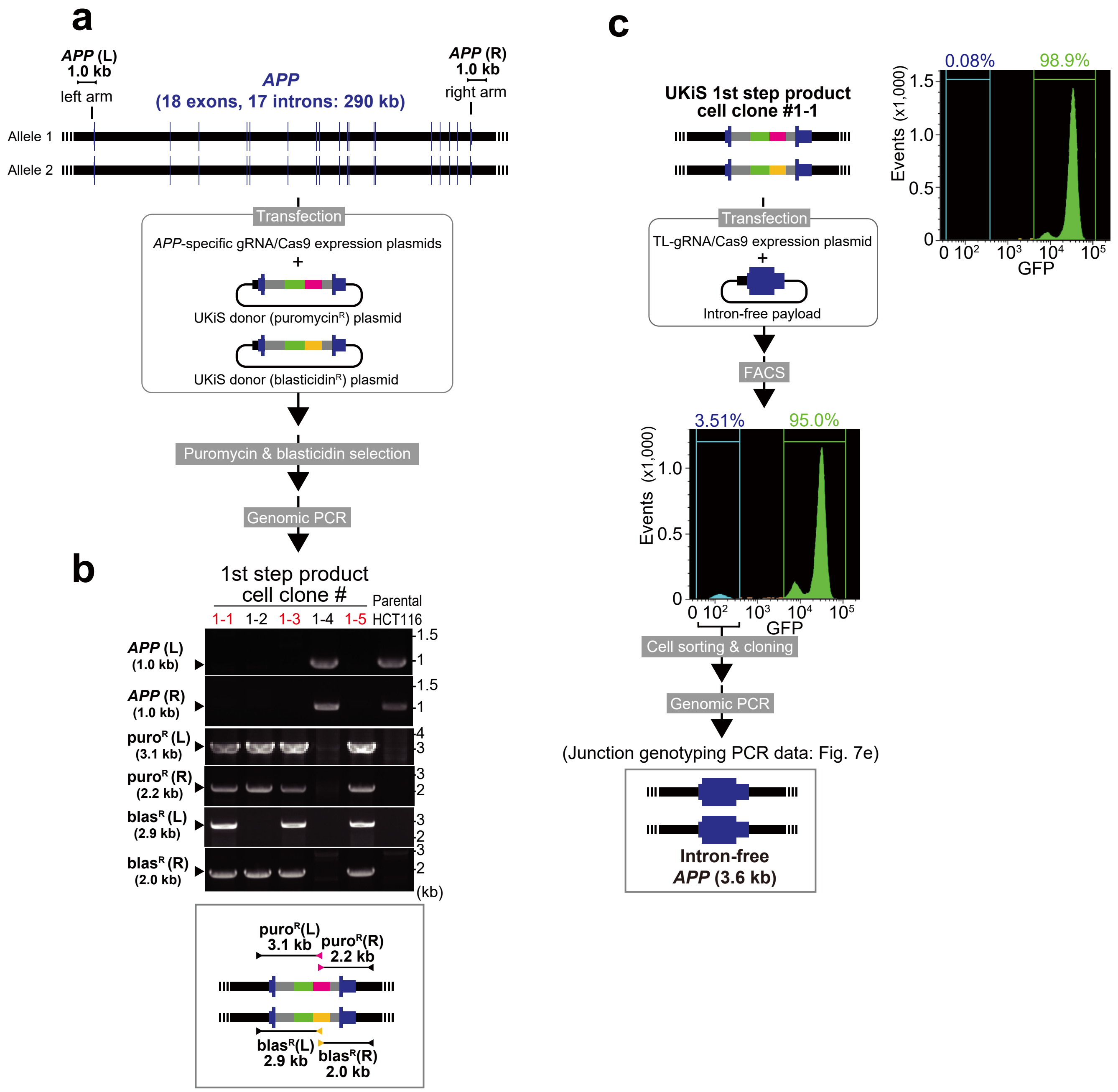

Supplementary Figure 15 | Deletion of all introns from *APP* in HCT116 cells by UKiS.

a. Schematic diagram of the first step of UKiS used to generate intron-free *APP* cell clones. Both UKiS donor plasmids and two *APP*-specific gRNA/Cas9 expression plasmids were transfected into HCT116 cells, leading to isolation of cell clones that underwent homologous recombination within the *APP* locus after dual selection with puromycin and blasticidin. Horizontal lines flanked by two arrowheads represent the target regions for junction genotyping PCR, with the expected length of PCR genotyping amplicons indicated.

b. Representative gel image of the junction genotyping PCR to confirm deletion of *APP* and insertion of the UKiS markers. Of the five selected clones, three (shown in red) had successful replacement of the *APP* sequence with the UKiS donor in both alleles. Source data are provided as a Source Data file.

c. Schematic diagram of the second step of UKiS during which both UKiS donor alleles were replaced with the intron-free *APP* sequence. First, the intron-free *APP* payload plasmid and the TL-gRNA/Cas9 expression plasmid were transfected into clone #1-1, which was obtained in (b) and mostly GFP-positive (98.9%) as shown by FACS analysis. Thereafter, GFP-negative cells were collected by FACS and cloned. Biallelic substitution of UKiS markers with the mutating payload plasmid was confirmed by junction genotyping PCR (Supplementary Fig. 18). A representative image of the resulting agarose gel electrophoresis of junction PCR amplicons is shown in Figure 7e.

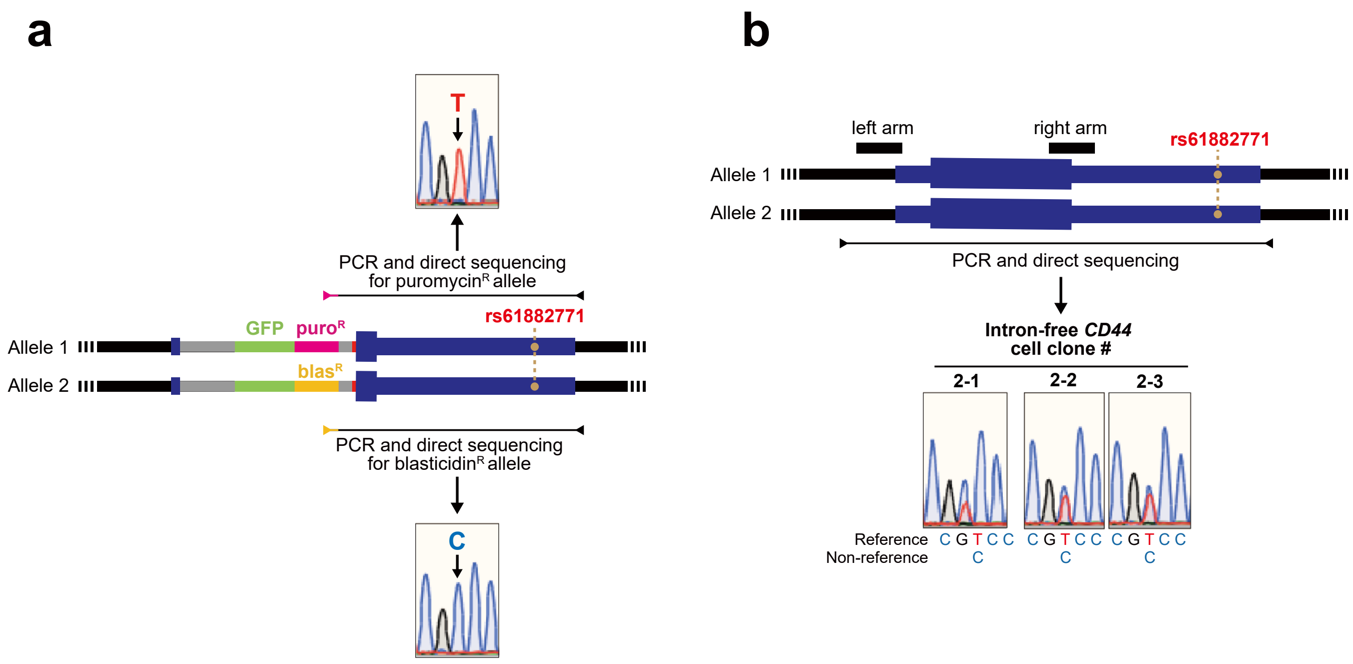

Supplementary Figure 16 | Validation of biallelic replacement of the *CD44* locus in the first and second step of UKiS to generate intron-free *CD44* cell clones.

a. Genotyping of clone #1-1 in the *CD44* region after the first step of UKiS. Clone #1-1 (Supplementary Fig. 13b) was then used to create the intron-free *CD44* cell clones. Allele-specific PCR was performed by using primers for puromycin or blasticidin marker sequences, demonstrating that the puromycin and blasticidin alleles have a T and C at rs61882771, respectively. Horizontal lines flanked by two arrowheads represent the target region for the PCR of each allele.

b. Graphical representation of the intron-free *CD44* locus is shown on the top: black boxes represent the positions of homology arms used in our UKiS mutagenesis to *CD44*, the horizontal line flanked by two arrowheads represents the target region for PCR, and the orange filled circles denote the positions of the heterozygous SNP site, rs61882771. Direct sequencing of the PCR genotyping amplicons indicated double peaks only at rs61882771 on the resultant sequencing chromatograms for all three clones of this mutant.

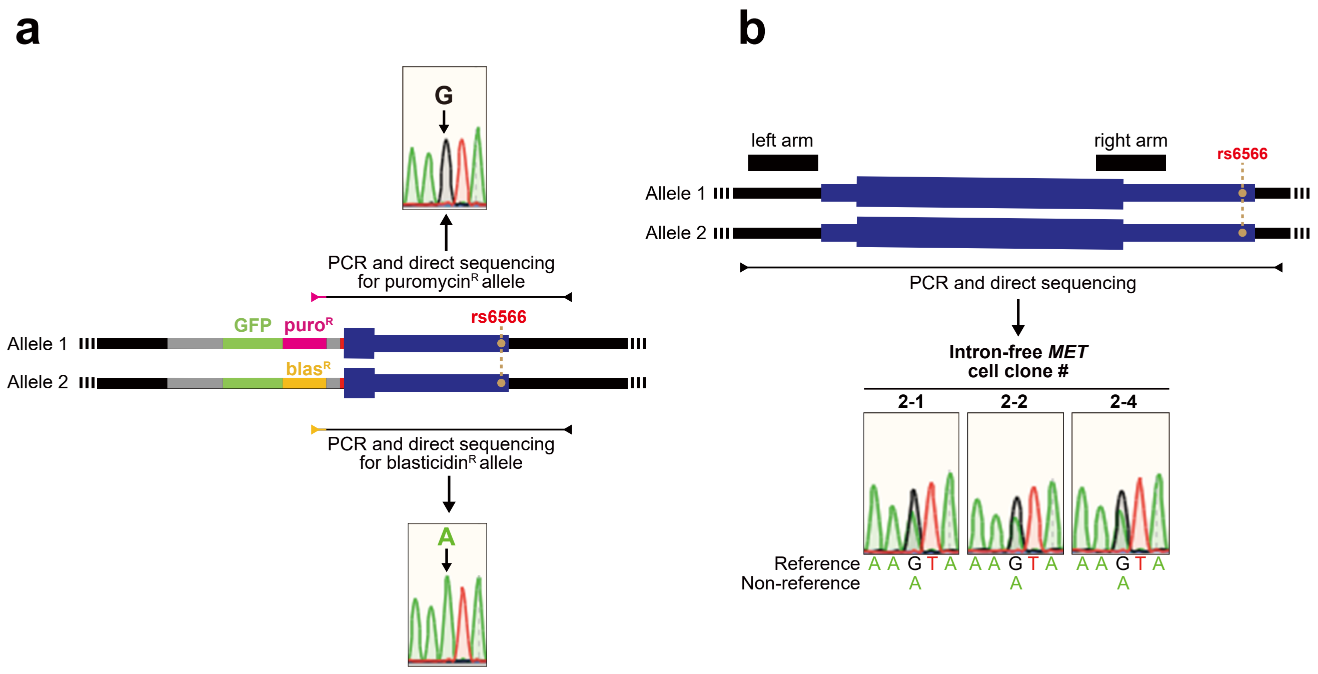

Supplementary Figure 17 | Validation of biallelic replacement of the *MET* locus in the first and second step of UKiS to generate intron-free *MET* cell clones.

a. Genotyping of clone #1-1 in the *MET* region after the first step of UKiS (Supplementary Fig. 14b). Clone #1-1 was then used to create the intron-free *MET* cell clones. Allele-specific PCR was performed by using primers for puromycin or blasticidin marker sequences, demonstrating that the puromycin and blasticidin alleles have a G and A at rs6566, respectively. Horizontal lines flanked by two arrowheads represent the target region for the PCR of each allele.

b. Graphical representation of the intron-free *MET* locus is shown on the top: black boxes represent the positions of homology arms used in our UKiS mutagenesis to *MET*, the horizontal line flanked by two arrowheads represents the target region for PCR, and the orange filled circles denote the positions of the heterozygous SNP site, rs6566. Direct sequencing of the PCR genotyping amplicons indicated double peaks only at rs6566 on the resultant sequencing chromatograms for all three clones of this mutant.

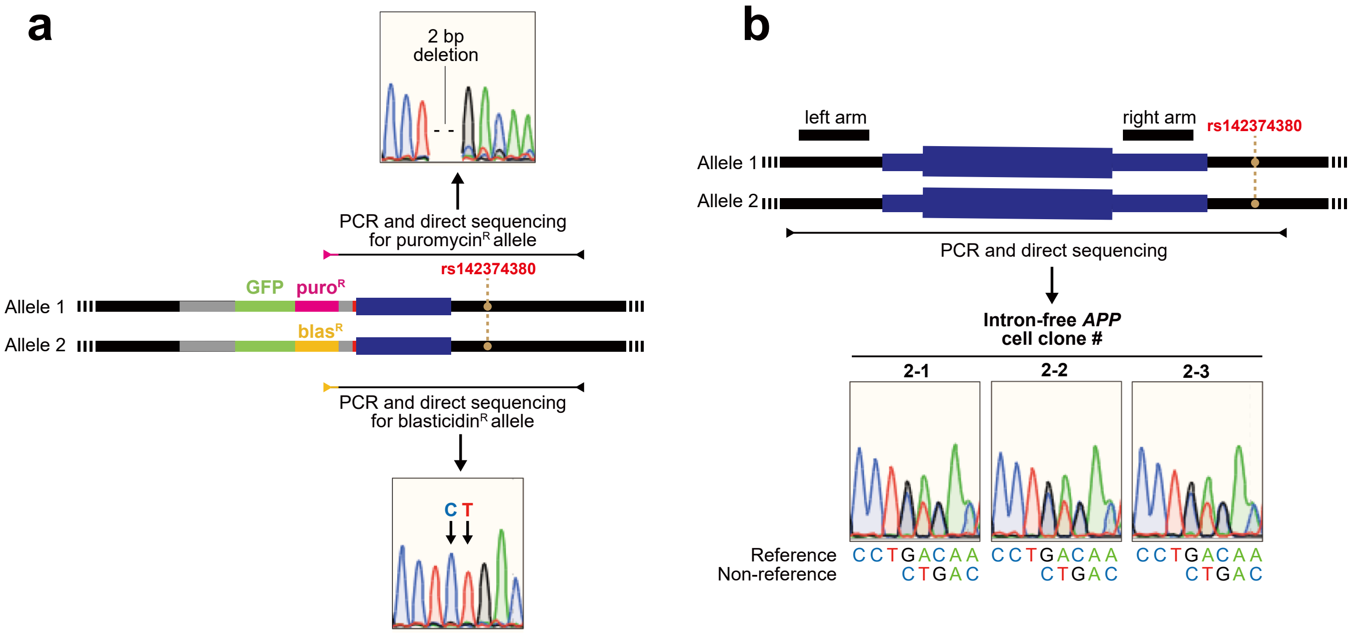

Supplementary Figure 18 | Validation of biallelic replacement of the *APP* locus in the first and second step of UKiS to generate intron-free *APP* cell clones.

a. Genotyping of clone #1-1 in the *APP* region after the first step of UKiS (Supplementary Fig. 15b). Clone #1-1 was then used to create the intron-free *APP* cell clones. Allele-specific PCR was performed by using primers for puromycin or blasticidin marker sequences, demonstrating that the puromycin and blasticidin alleles have a 2-bp deletion and a CT at rs142374380, respectively. Horizontal lines flanked by two arrowheads represent the target region for the PCR of each allele.

b. Graphical representation of the intron-free *APP* gene locus is shown on the top: black boxes represent the positions of homology arms used in our UKiS mutagenesis to *APP*, the horizontal line flanked by two arrowheads represents the target region for PCR, and the orange filled circles denote the positions of the heterozygous SNP site, rs142374380. Direct sequencing of the PCR genotyping amplicons indicated an equivalent mixture of the 2-bp deletion and CT peaks at rs142374380 on the resultant sequencing chromatograms for all three clones of this mutant.

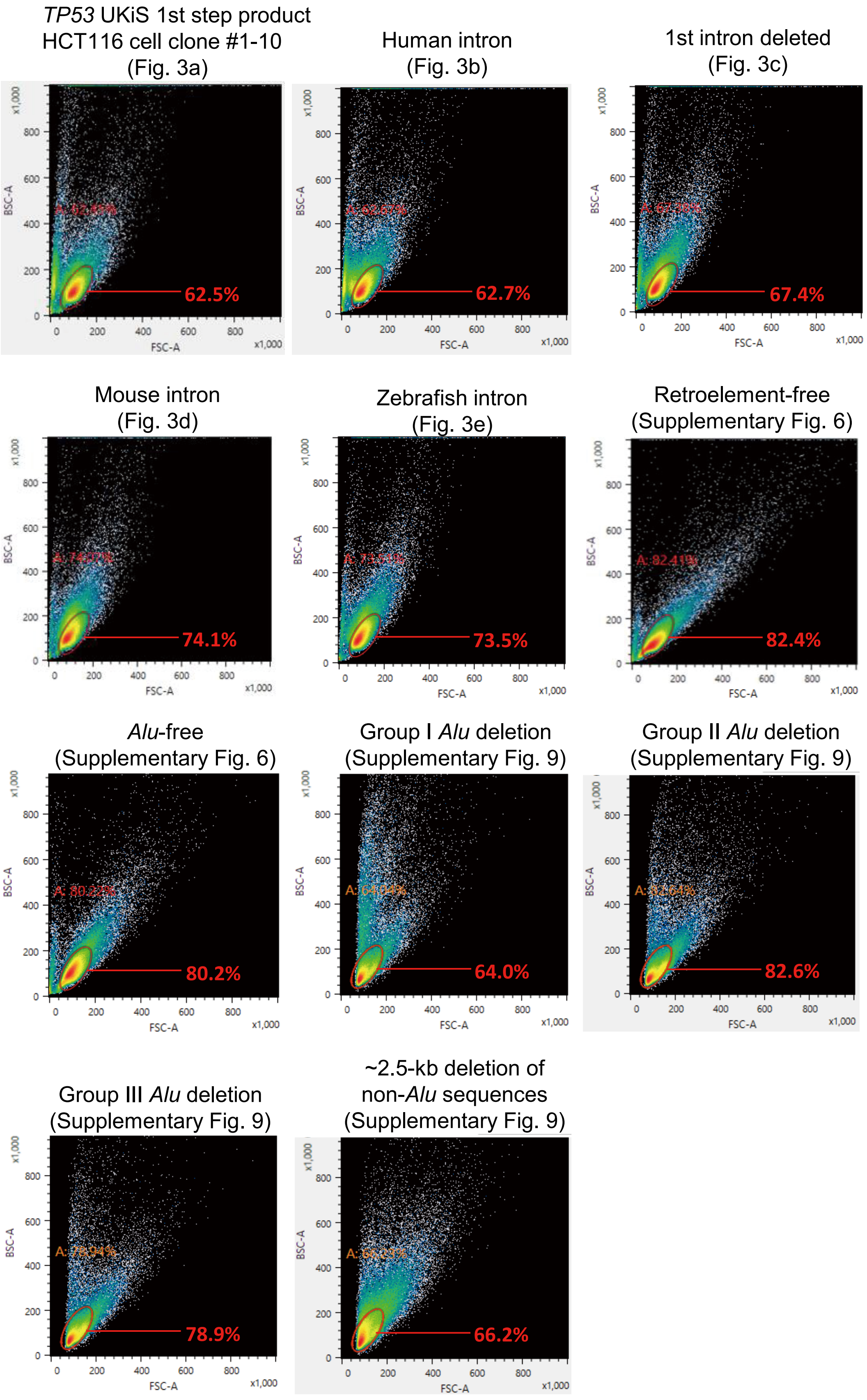

Supplementary Figure 19 | Gating strategies used at the UKiS second step of *TP53* 1st intron mutagenesis in HCT116 cells by Cell Sorter SH800.

Cells in the areas surrounded by red ellipses in the FSC-A vs BSC-A plots were analyzed and/or sorted depending on the green fluorescence intensities as shown in the figures (Figs. 3a-e and Supplementary Figs. 6 and 9).

TP53 UKiS 1st step product iPS cell clone #1-2 (Supplementary Fig. 11c)

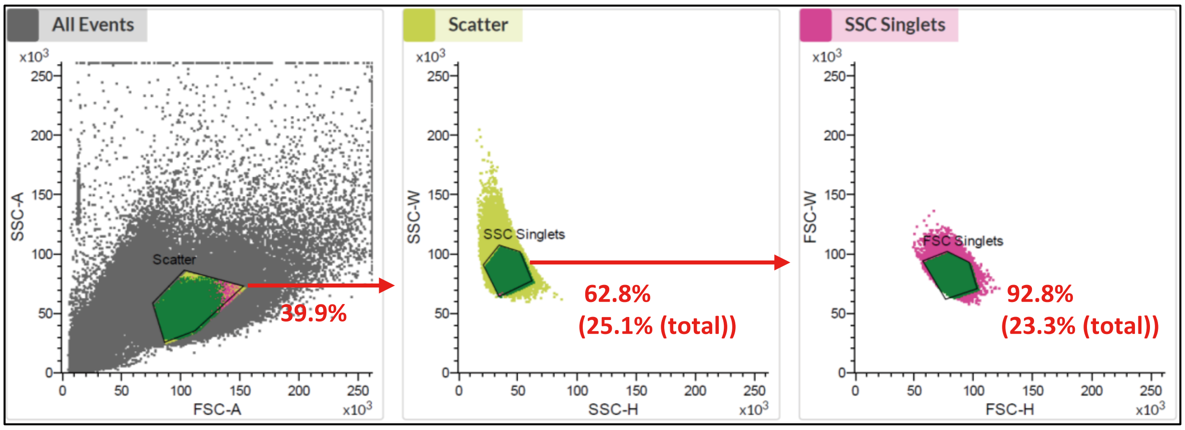

TP53 1st intron deleted iPS cell (Supplementary Fig. 11c)

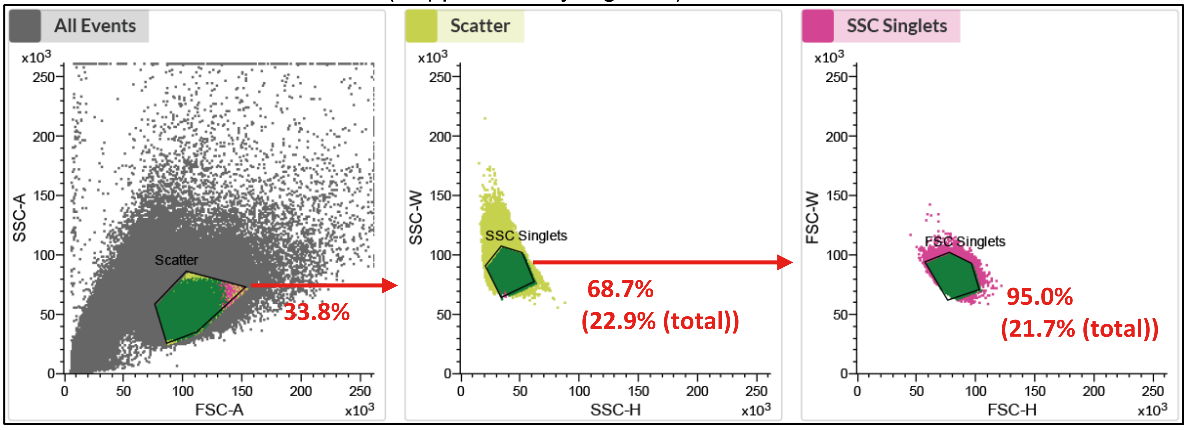

Supplementary Figure 20 | Gating strategies used at the UKiS second step of *TP53* 1st intron deletion in iPS cells by BD FACSMelody Cell Sorter.
Cells in the areas surrounded by black lines in the FSC-A vs SSC-A plots (left) were selected to remove cell debris, which were then subjected to two more filtering steps (SSC-H vs SSC-W (middle) and FSC-H vs FSC-W (right)) to select single isolated cells. The resultant cell populations were analyzed and/or sorted depending on the green fluorescence intensities, as shown in the figure (Supplementary Fig. 11c).

CD44 UKiS 1st step product HCT116 cell clone #1-1 (Supplementary Fig. 13c)

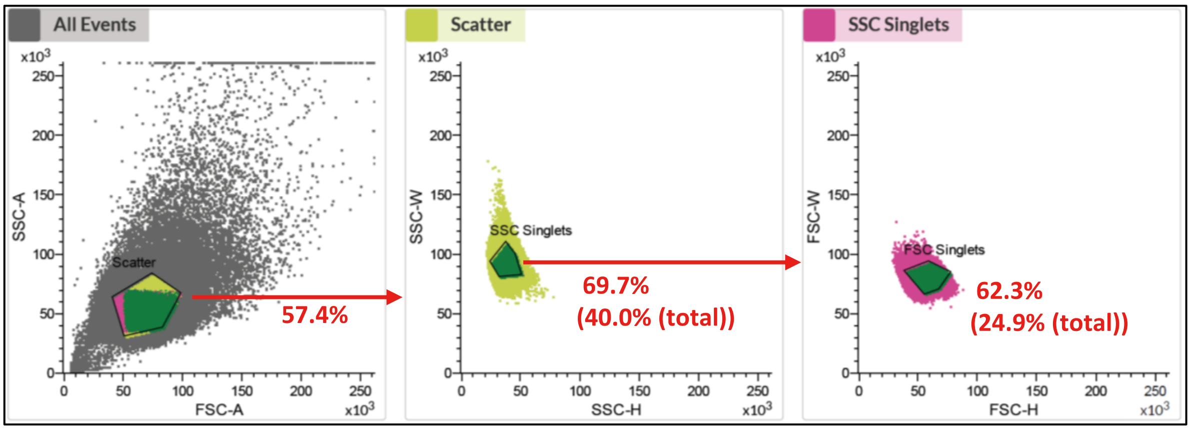

Intron-free *CD44* HCT116 cell (Supplementary Fig. 13c)

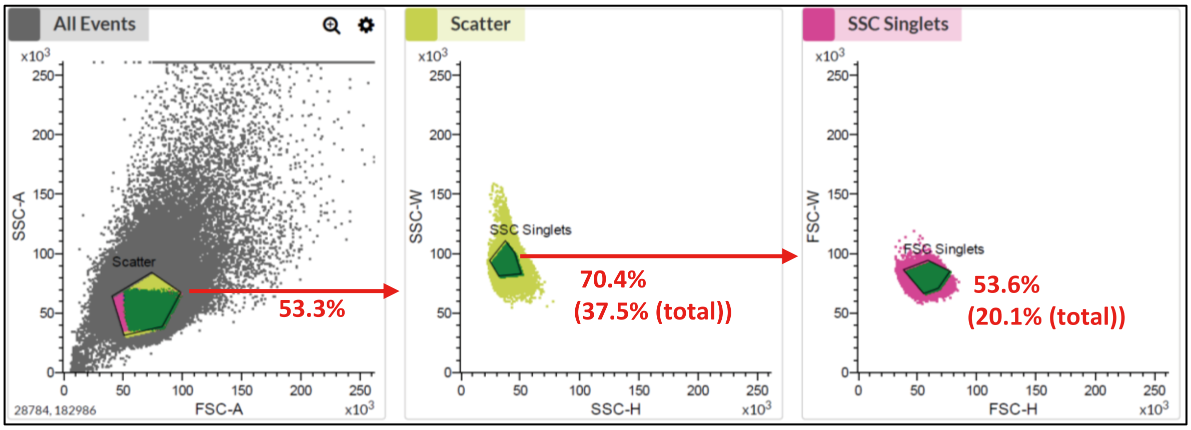

MET UKiS 1st step product HCT116 cell clone #1-1 (Supplementary Fig. 14c)

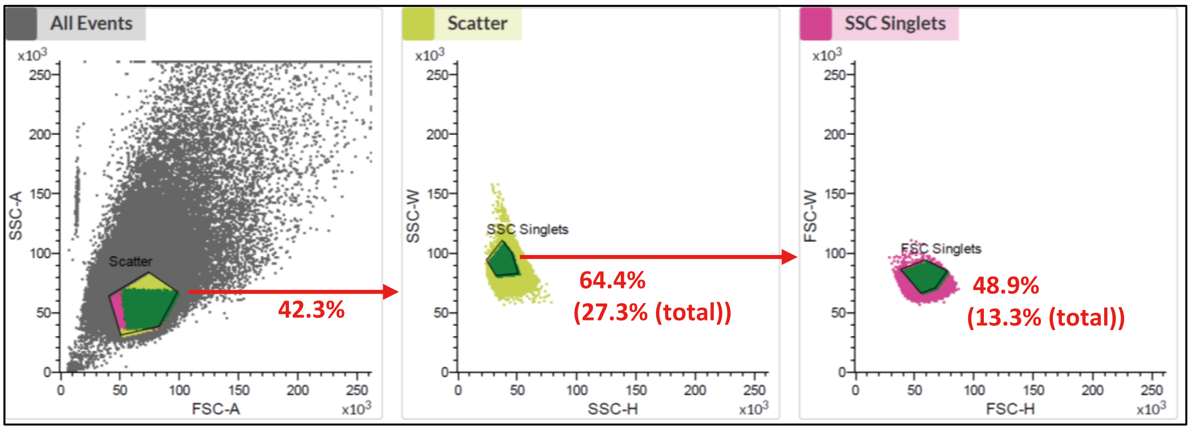

Supplementary Figure 21 | Gating strategies used at the UKiS second steps of *CD44*, *MET* and *APP* all-intron deletion in HCT116 cells by BD FACSMelody Cell Sorter.
Cells in the areas surrounded by black lines in the FSC-A vs SSC-A plots (left) were selected to remove cell debris, which were then subjected to two more filtering steps (SSC-H vs SSC-W (middle) and FSC-H vs FSC-W (right)) to select single isolated cells. The resultant cell populations were analyzed and/or sorted depending on the green fluorescence intensities, as shown in the figures (Supplementary Figs. 13c, 14c and 15c).

(Continued)

Intron-free *MET* HCT116 cell (Supplementary Fig. 14c)

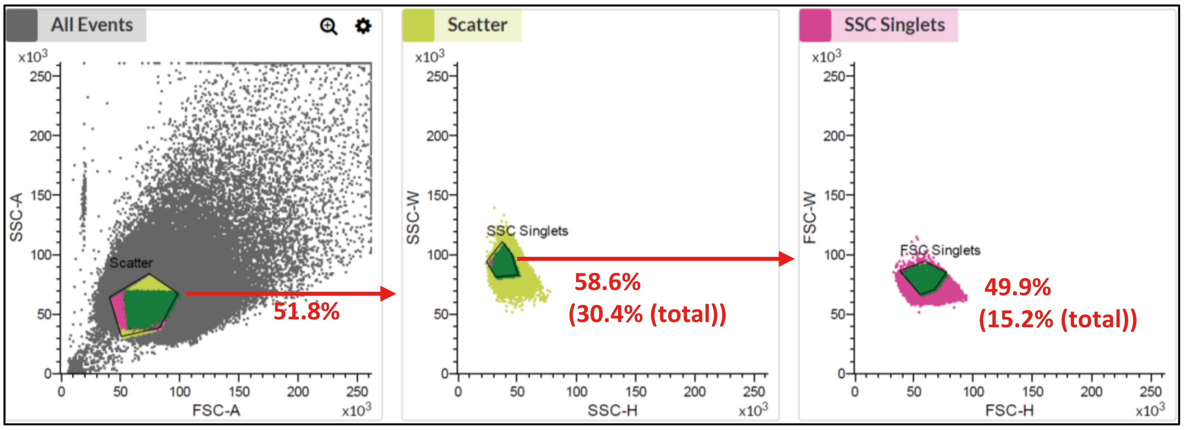

APP UKiS 1st step product HCT116 cell clone #1-1 (Supplementary Fig. 15c)

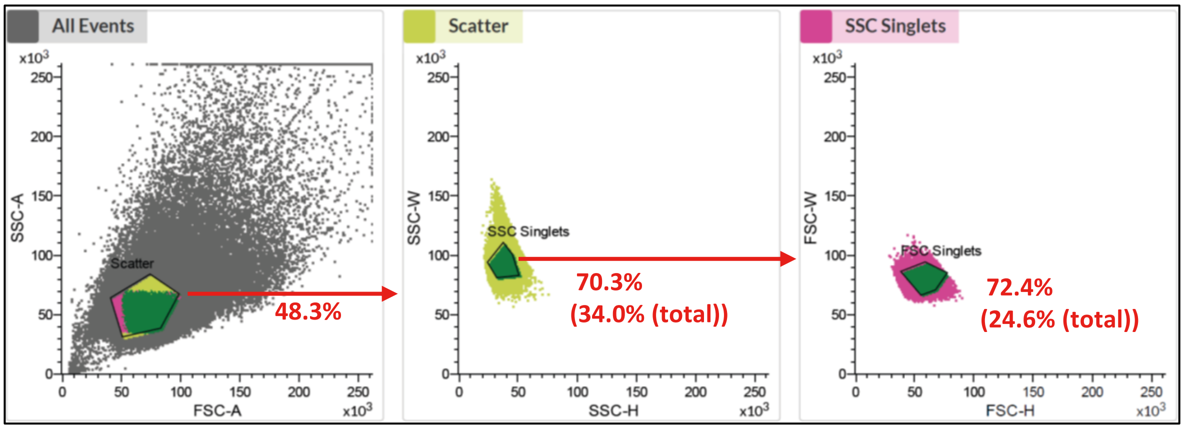

Intron-free *APP* HCT116 cell (Supplementary Fig. 15c)

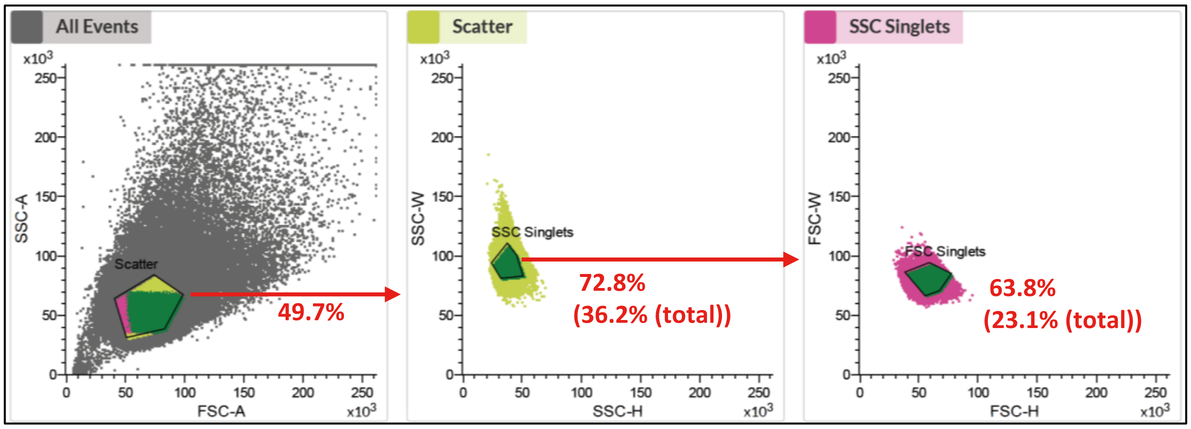

Supplementary Table 1. Primers used for genotyping junction PCR.

Fig. #	Target	Forward primer	Reverse primer
Fig. 2c Supplementary Figs. 2b and 11b	*TP53* (L)	TGCCCCGTTGTTATCCTTAC	GATGCAGAAGAGGTGCAAGA
	TP53 (R)	GAGCCGCAGTCAGATCCTAG	GAAGACGGCAGCAAAGAAAC
	puro^R (L) (*TP53*)	TGCCCCGTTGTTATCCTTAC	GCTCGTAGAAGGGGAGGTTG
	puro^R (R) (*TP53*)	GTCACCGAGCTGCAAGAAC	GAAGACGGCAGCAAAGAAAC
	blas^R (L) (*TP53*)	TGCCCCGTTGTTATCCTTAC	GCTTCAATATGTACTGCCGAAA
	blas^R (R) (*TP53*)	GAAGCCATTGCGATTGGTAG	GAAGACGGCAGCAAAGAAAC
Figs. 3b-e and 7a Supplementary Figs. 6 and 9	eintire *TP53* first intron	TGCCCCGTTGTTATCCTTAC	GAAGACGGCAGCAAAGAAAC
	UKiS(L) (*TP53*)	TGCCCCGTTGTTATCCTTAC	GGGCTGTCCCTCATAAAAGT
	UKiS(R) (*TP53*)	TCACGTAAGTAGAACATGAAATAACC	GAAGACGGCAGCAAAGAAAC
Fig. 7c	entire *CD44*	AGTGGATGGACAGGAGGATG	GAGTGGGTCTGAGTGGGAAC
Fig. 7d	entire *MET*	TGAAATCACTCTTATGTAACCTCTGG	TGCAGGTATAGGCAGTGACAAG
Fig. 7e	entire *APP*	GGGGAGCTGGTACAGAAATG	CTCTTCTCCCCACCCAAAA
Supplementary Fig. 13b	*CD44* (L)	AGTGGATGGACAGGAGGATG	ACCCATCTTGCTGCCCAGG
	CD44 (R)	ATGGAGCTGTGGAGGACAGA	GAGTGGGTCTGAGTGGGAAC
	puro^R (L) (*CD44*)	AGTGGATGGACAGGAGGATG	GCTCGTAGAAGGGGAGGTTG
	puro^R (R) (*CD44*)	GTCACCGAGCTGCAAGAAC	GAGTGGGTCTGAGTGGGAAC
	blas^R (L) (*CD44*)	AGTGGATGGACAGGAGGATG	GCTTCAATATGTACTGCCGAAA
	blas^R (R) (*CD44*)	GAAGCCATTGCGATTGGTAG	GAGTGGGTCTGAGTGGGAAC
Supplementary Fig. 14b	*MET* (L)	TGAAATCACTCTTATGTAACCTCTGG	CCGGGCATCGGCGCGCGCGGCCC
	MET (R)	ACCCACTGTTTGAGAATGATG	TGCAGGTATAGGCAGTGACAAG
	puro^R (L) (*MET*)	TGAAATCACTCTTATGTAACCTCTGG	GCTCGTAGAAGGGGAGGTTG
	puro^R (R) (*MET*)	GTCACCGAGCTGCAAGAAC	TGCAGGTATAGGCAGTGACAAG
	blas^R (L) (*MET*)	TGAAATCACTCTTATGTAACCTCTGG	GCTTCAATATGTACTGCCGAAA
	blas^R (R) (*MET*)	GAAGCCATTGCGATTGGTAG	TGCAGGTATAGGCAGTGACAAG
Supplementary Fig. 15b	*APP* (L)	GGGGAGCTGGTACAGAAATG	CAGGATCAGGGAAAGGTGAG
	APP (R)	GAACGGCTACGAAAATCCAA	CTCTTCTCCCCACCCAAAA
	puro^R (L) (*APP*)	GGGGAGCTGGTACAGAAATG	GCTCGTAGAAGGGGAGGTTG
	puro^R (R) (*APP*)	GTCACCGAGCTGCAAGAAC	CTCTTCTCCCCACCCAAAA
	blas^R (L) (*APP*)	GGGGAGCTGGTACAGAAATG	GCTTCAATATGTACTGCCGAAA
	blas^R (R) (*APP*)	GAAGCCATTGCGATTGGTAG	CTCTTCTCCCCACCCAAAA

Supplementary Table 2. Primers used for SNP typing.

Primers used for PCR

Fig. #	Target	Forward primer	Reverse primer
Fig. 4a	From *TP53* 4th intron to 7th intron	GGCTTTTATCCATCCCATCA	AGCTGTTCCGTCCCAGTAGA
Fig. 4b	From puromycin resistance gene sequence to *TP53* 7th intron	GTCACCGAGCTGCAAGAAC	AGCTGTTCCGTCCCAGTAGA
	From blasticidin resistance gene sequence to *TP53* 7th intron	GAAGCCATTGCGATTGGTAG	AGCTGTTCCGTCCCAGTAGA
Fig. 4c<br>Supplementary Figs. 5, 7 and 10	From upstream of *TP53* 1st exon to 7th intron	TGCCCCGTTGTTATCCTTAC	AGCTGTTCCGTCCCAGTAGA
Supplementary Fig. 12a	From puromycin resistance gene sequence to *TP53* 11th intron	GTCACCGAGCTGCAAGAAC	AGCTGCCTTTGACCATGAAG
	From blasticidin resistance gene sequence to *TP53* 11th intron	GAAGCCATTGCGATTGGTAG	AGCTGCCTTTGACCATGAAG
Supplementary Fig. 12b	From upstream of *TP53* 1st exon to 10th intron	TGCCCCGTTGTTATCCTTAC	AGCTGCCTTTGACCATGAAG
Supplementary Fig. 16a	From puromycin resistance gene sequence to downstream of *CD44* last exon	GTCACCGAGCTGCAAGAAC	AAGTTGATGGAAACCCATTTTG
	From blasticidin resistance gene sequence to downstream of *CD44* last exon	GAAGCCATTGCGATTGGTAG	AAGTTGATGGAAACCCATTTTG
Supplementary Fig. 16b	From upstream of *CD44* 1st exon to downstream of last exon	AGTGGATGGACAGGAGGATG	AAGTTGATGGAAACCCATTTTG
Supplementary Fig. 17a	From puromycin resistance gene sequence to downstream of *MET* last exon	GTCACCGAGCTGCAAGAAC	CCAAACCCTTGGAAAGACAG
	From blasticidin resistance gene sequence to downstream of *MET* last exon	GAAGCCATTGCGATTGGTAG	CCAAACCCTTGGAAAGACAG
Supplementary Fig. 17b	From upstream of *MET* 1st exon to downstream of last exon	TGAAATCACTCTTATGTAACCTCTGG	CCAAACCCTTGGAAAGACAG
Supplementary Fig. 18a	From puromycin resistance gene sequence to downstream of *APP* last exon	GTCACCGAGCTGCAAGAAC	ATTTGGCAGCACTTCAGTTT
	From blasticidin resistance gene sequence to downstream of APP last exon	GAAGCCATTGCGATTGGTAG	ATTTGGCAGCACTTCAGTTT
Supplementary Fig. 18b	From upstream of *APP* 1st exon to downstream of last exon	GGGGAGCTGGTACAGAAATG	ATTTGGCAGCACTTCAGTTT

Primers used for direct sequencing

Fig. #	Target	Primer
Fig. 4a	Heterozygous SNP candidates in *TP53* locus (rs1794287, rs9895829 and rs2909430)	GGGATTCCTCTTCACCCTTT
	Heterozygous SNP candidates in *TP53* locus (rs1800372 and rs1625895)	AGGTGTAGACGCCAACTCTCTC
	Heterozygous SNP candidates in *TP53* locus (rs8069054 and rs17880604)	GTGTGGTGGTGCCCTATGA
Fig. 4a, 4b and 4c<br>Supplementary Figs. 5, 7, 10	rs12947788	AGCTGTTCCGTCCCAGTAGA
Supplementary Fig. 12	rs1641548	AGCTGCCTTTGACCATGAAG
Supplementary Fig. 16	rs61882771	CCATTTTCAGTGGTCTGGATT
Supplementary Fig. 17	rs6566	TCTGCTCTGTGGAAAGAAAGAA
Supplementary Fig. 18	rs142374380	ATTTGGCAGCACTTCAGTTT

Supplementary Table 3. Primers used for RT-PCR.

Fig. #	Target	Forward primer	Reverse primer
Fig. 5a	*TP53*	CTCAAGACTGGCGCTAAAAG	CTGCCCTGGTAGGTTTTCTG
Figs. 5a and 7f-h	18S rRNA	ATTAATCAAGAACGAAAGTCGGA GGT	TTTAAGTTTCAGCTTTGCAACCAT ACT
Fig. 7f	*CD44*	CATCCTCGTCCCGTCCTC	CCATGAGATTTGGCTGAGTG
Fig. 7g	*MET*	GAGCGCCTCAGTCTGGTC	TGCAGGTATAGGCAGTGACAAG
Fig. 7h	*APP*	GATCCCACTCGCACAGCA	TTCTCCCCACCCAAAATTAC

Supplementary Table 4. All primers and oligonucleotides used for plasmid construction and sequencing of constructed plasmid.

Primers and oligonucleotides used for plasmid construction

Plasmid	Description	Backbone	PCR target	PCR template	Forward primer	Reverse primer
HR110PA-1_GFP_puro	HR110PA-1 plasmid (RFP coding sequence of HR110PA-1 was replaced by GFP)	HR110PA-1	Left insulator, EF1 promoter and part of backbone	HR110PA-1	GGTGATGACGGTGAAAACCT	CCCTTGCTCACGGTACCCATGGTGGCGATATCGTAGGCG
			T2A, Puromycin resistance gene, right insulator and part of backbone	HR110PA-1	GAGGGCAGAGGAAGTCTTCT	AGGTTTTCACCGTCATCACC
			GFP	pEGFP-F (Clontech)	ATGGGTACCGTGAGCAAGG	CTCCACGTCACCGCATGTTAGAAGACTTCCTCTGCCCTCCTTGTACAGCTCGTCCATG
HR110PA-1_GFP_blas	HR110PA-1 plasmid (Puromycin resistance gene of HR110PA-1_GFP was replaced by that of blasticidin resistance gene)	HR110PA-1	Blasticidin resistance gene	pCISP310B	ATGAAAACATTTAACATTTCTCAAC	TTTTAATTTCGGGTATATTTGAGTGGA
			Left insulator, EF1 promoter, GFP and part of backbone	HR110PA-1_GFP_puro	GGTGATGACGGTGAAAACCT	AATGTTAAATGTTTTCATAGGGCCGGGATTCTCCTCCA
			T2A, right insulator and part of backbone	HR110PA-1_GFP_puro	AAATATACCCGAAATTAAAATCAACCTCTGGACGAAAT	AGGTTTTCACCGTCATCACC
pTO455	UKiS donor (puromycin resistance gene) for *TP53* locus	HR110PA-1	*TP53* left arm	HCT116 genome	GTAAAACGACGGCCAGTGAATTCTTCCCCAGAGGTATCTTCCA	AGCGTAGGCGCTTCTCGCCAAGAT
			UKiS marker sequence (puromycin resistance gene), from left insulator to T2A	HR110PA-1_GFP_puro	GAATTCGAGCTCGGTACCTC	AGGGCCGGGATTCTCCTCCAC
			UKiS marker sequence (puromycin resistance gene), from T2A to right insulator		GAGGGCAGAGGAAGTCTTCT	CCCTTACGCGATCGCGTTGC
			TP53 right arm	HCT116 genome	GCGCAACGCGATCGCGTAAGGGCTGAGTCAGGAAACATTTTCAG	CTTGCATGCAGTCGACGGGGATCCAAGTTCTGCATCCCCAGGAG
pTO456	UKiS donor (blasticidin resistance gene) for *TP53* locus	HR110PA-1	*TP53* left arm	HCT116 genome	GTAAAACGACGGCCAGTGAATTCTTCCCCAGAGGTATCTTCCA	AGCGTAGGCGCTTCTCGCCAAGAT
			UKiS marker sequence (puromycin resistance gene), from left insulator to T2A	HR110PA-1_GFP_blas	GAATTCGAGCTCGGTACCTC	AGGGCCGGGATTCTCCTCCAC
			UKiS marker sequence (puromycin resistance gene), from T2A to right insulator		GAGGGCAGAGGAAGTCTTCT	CCCTTACGCGATCGCGTTGC
			TP53 right arm	HCT116 genome	GCGCAACGCGATCGCGTAAGGGCTGAGTCAGGAAACATTTTCAG	CTTGCATGCAGTCGACGGGGATCCAAGTTCTGCATCCCCAGGAG
pTO467	Mutating payload for creating human *TP53* 1st intron restored cell	HR110PA-1	*TP53* left arm to 1st exon	HCT116 genome	GTAAAACGACGGCCAGTGAATTCTTCCCCAGAGGTATCTTCCA	CCAATCCAGGGAAGCGTGT
			TP53 2nd exon to right arm	HCT116 genome	CAGCCAGACTGCCTTCCGG	CTTGCATGCAGTCGACGGGGATCCAAGTTCTGCATCCCCAGGAG
			Human *TP53* 1st intron	HCT116 genome	GTAAAACGACGGCCAGTGAATTCTTCCCCAGAGGTATCTTCCA	CTTGCATGCAGTCGACGGGGATCCAAGTTCTGCATCCCCAGGAG
pTO469	Mutating payload for creating mouse *Tp53* 1st intron replaced cell	HR110PA-1	*TP53* left arm to 1st exon	HCT116 genome	GTAAAACGACGGCCAGTGAATTCTTCCCCAGAGGTATCTTCCA	CCAATCCAGGGAAGCGTGT
			TP53 2nd exon to right arm	HCT116 genome	CAGCCAGACTGCCTTCCGG	CTTGCATGCAGTCGACGGGGATCCAAGTTCTGCATCCCCAGGAG
			Mouse *Tp53* 1st intron	Mouse genome	ACACGCTTCCCTGGATTGGGTAAGTAATTGATGAGCGTGA	CGGAAGGCAGTCTGGCTGCTGTAGAGAGAAGAGATTGTG
pTO470	Mutating payload for creating zebrafish *tp5*3 1st intron replaced cell	HR110PA-1	*TP53* left arm to 1st exon	HCT116 genome	GTAAAACGACGGCCAGTGAATTCTTCCCCAGAGGTATCTTCCA	CCAATCCAGGGAAGCGTGT
			TP53 2nd exon to right arm	HCT116 genome	CAGCCAGACTGCCTTCCGG	CTTGCATGCAGTCGACGGGGATCCAAGTTCTGCATCCCCAGGAG
			Zebrafish *tp53* 1st intron	Zebrafish genome	ACACGCTTCCCTGGATTGGGTAACAAAGCGAAACTTTTATTG	CCGGAAGGCAGTCTGGCTGCTGTGAAATTATAAACACGAAAG
pTO471	Mutating payload for creating *TP53* 1st intron deleted cell	HR110PA-1	*TP53* left arm to 1st exon	HCT116 genome	GTAAAACGACGGCCAGTGAATTCTTCCCCAGAGGTATCTTCCA	CCAATCCAGGGAAGCGTGT
			TP53 2nd exon to right arm	HCT116 genome	CAGCCAGACTGCCTTCCGG	CTTGCATGCAGTCGACGGGGATCCAAGTTCTGCATCCCCAGGAG
			From *TP53* 1st exon to 2nd exon excluding 1st intron sequence	HCT116 cDNA	GATGGGATTGGGGTTTTCCC	CCAATCCAGGGAAGCGTGTC

(Continued)

pTO468	Mutating payload for creating retroelement-free *TP53* 1st intron cell	HR110PA-1	Retroelement-free fragment #1 including left arm	pTO467	GTAAAACGACGGCCAGTGAATTCTTCCCCAGAGGTATCTTCCA	TTTAGGAAAAAGATGACGTAAGTACG
			Retroelement-free fragment #2		CGTACTTACGTCATCTTTTTCCTAAACACTGGACATATAGGCCTTC	CTAGTACTCTGTGTATTATGGGAATTCTGCAATTGTTCTATTTCACTTG
			Retroelement-free fragment #3		CCCATAATACACAGAGTACTAGGTAAAAGAAAAGAGCCTGCCATT	CTACTGTAAGTGTTTGTTACACCACTTTTCTGTTGTTTGCACTG
			Retroelement-free fragment #4		GTGCAAACAACAGAAAAGTGGTGTAACAAACACTTACAGTAG	CAAAGGCTTTGCCATGTTTCGCCATGAGGATGCTCTCTTTCTTTTTC
			Retroelement-free fragment #5		GAAAGAGAGCATCCTCATGGCGAAACATGGCAAAGCCTTTGAAAG	GTCATCCTATTTTAATTCACATCAGAATAACACACAAGCCTGTTATA
			Retroelement-free fragment #6		AACAGGCTTGTGTGTTATTCTGATGTGAATTAAAATAGGATGAC	TCCTGTGGAGCAGGAAAAGATCCCACCTCCTGTTAACAAGG
			Retroelement-free fragment #7 including right arm		CCTTGTTAACAGGAGGTGGGATCTTTTCCTGCTCCACAGGA	CTTGCATGCAGTCGACGGGGATCCAAGTTCTGCATCCCCAGGAG
pTO504	Mutating payload for creating *Alu*-free *TP53* 1st intron cell	HR110PA-1	*Alu*-free fragment #1 including left HA arm	pTO467	GTAAAACGACGGCCAGTGAATTCTTCCCCAGAGGTATCTTCCA	CACATAATGTATGATTCCCCCCTGATTCCATTCTATATGAAGTTCTCC
			Alu-free fragment #2		GGAGAACTTCATATAGAATGGAATCAGGGGGGAATCATACATTATGTG	GGAGATAAATAGAAAATAGCACTAAGATAGCACTAAAATGGTAGCCCTA
			Alu-free fragment #3		TAGGGCTACCATTTTAGTGCTATCTTAGTGCTATTTTCTATTTATCTCC	AACAAGTTGGGAAATATTCCTCTATGTTCCTCTGTTTTCTGAAGAG
			Alu-free fragment #4		CTCTTCAGAAAACAGAGGAACATAGAGGAATATTTCCCAACTTGTT	TAAACATTATTTGGTTAGAATGTATATTTATCCTTTTTTCTATATTACC
			Alu-free fragment #5		GGTAATATAGAAAAAAGGATAAATATACATTCTAACCAAATAATGTTTA	TCTTAGAGGAAAAAGCGTTGAGTTAGCACTGAGTTTTCTACCATTA
			Alu-free fragment #6		TAATGGTAGAAAACTCAGTGCTAACTCAACGCTTTTTCCTCTAAGA	TATTTCTTCCTTTCTAATCGTTATTACTAATCACTCATTATTTCTTTTTCTTGCCTG
			Alu-free fragment #7		CAGGCAAGAAAAAGAAATAATGAGTGATTAGTAATAACGATTAGAAAGGAAGAAATA	TTTTTGAGATAGAGTTTCACCCATTAGGACATGTATGTATAGAA
			Alu-free fragment #8		TTCTATACATACATGTCCTAATGGGTGAAACTCTATCTCAAAAA	CTTTAATGGCAGGCTCTTTTCTTTTACCTAGTACTCTGTGTATTATGGG
			Alu-free fragment #9		CCCATAATACACAGAGTACTAGGTAAAAGAAAAGAGCCTGCCATTAAAG	CCTACTGTAAGTGTTTGTTACACCACTTTTCTGTTGTTTGCACTGA
			Alu-free fragment #10		TCAGTGCAAACAACAGAAAAGTGGTGTAACAAACACTTACAGTAGG	TCAAAGGCTTTGCCATGTTTCGCCATGAGGATGCTCTCTTTCTTTTTCT
			Alu-free fragment #11		AGAAAAAGAAAGAGAGCATCCTCATGGCGAAACATGGCAAAGCCTTTGA	TTCCCAACTCCCTTCCTGTATCCCACCTCCTGTTAACAAG
			Alu-free fragment #12 including right HA arm		CTTGTTAACAGGAGGTGGGATACAGGAAGGGAGTTGGGAA	CTTGCATGCAGTCGACGGGGATCCAAGTTCTGCATCCCCAGGAG
pTO642	Mutating payload for creating ~2.5-kb non-*Alu* sequences deleted *TP53* 1st intron cell	HR110PA-1	Non-*Alu* sequence deleted fragment #1	pTO467	GTAAAACGACGGCCAGTGAATTCTTCCCCAGAGGTATCTTCCA	TTAAAATTAAAAGCTTTTTTTTGAGACGGAGTTTCGTTCTTGT
			Non-*Alu* sequence deleted fragment #2	pTO467	TCTCAAAAAAAAGCTTTTAATTTTAATTATTTTACAGTTGGAG	GCTCAACAAAGGTTAGCTCTATATTGCCCTGTAACCTGCAA
			Non-*Alu* sequence deleted fragment #3	pTO467	AGAGCTAACCTTTGTTGAGC	TCAAAGGCTTTGCCATGTTT
			Non-*Alu* sequence deleted fragment #4	pTO467	AAACATGGCAAAGCCTTTGACTTGTTAACAGGAGGTGGGA	CTTGCATGCAGTCGACGGGGATCCAAGTTCTGCATCCCCAGGAG
pTO643	Mutating payload for Group I *Alu* sequences deleted *TP53* 1st intron cell	HR110PA-1	Sequence from left HA arm to downstream of group I *Alu* excluding *Alu* sequnces	pTO504	GTAAAACGACGGCCAGTGAATTCTTCCCCAGAGGTATCTTCCA	AGCACTGAGTTTTCTACCATTATG
			Sequence from downstream of group I *Alu* to right HA arm	pTO467	CATAATGGTAGAAAACTCAGTGCT	CTTGCATGCAGTCGACGGGGATCCAAGTTCTGCATCCCCAGGAG
pTO644	Mutating payload for Group II *Alu* sequences deleted *TP53* 1st intron cell	HR110PA-1	Sequence from left HA arm to downstream of group I *Alu*	pTO467	GTAAAACGACGGCCAGTGAATTCTTCCCCAGAGGTATCTTCCA	AGCACTGAGTTTTCTACCATTATG
			Sequence from downstream of group I *Alu* to downstream of group II *Alu* excluding *Alu* sequences	pTO504	CATAATGGTAGAAAACTCAGTGCT	CCTACAGATAGAGGGATAACT
			Sequence from downstream of group II *Alu* to right HA arm	pTO467	AGTTATCCCTCTATCTGTAGG	CTTGCATGCAGTCGACGGGGATCCAAGTTCTGCATCCCCAGGAG
pTO645	Mutating payload for Group III *Alu* sequences deleted *TP53* 1st intron cell	HR110PA-1	Sequence from left HA arm to downstream of group II *Alu*	pTO467	GTAAAACGACGGCCAGTGAATTCTTCCCCAGAGGTATCTTCCA	CCTACAGATAGAGGGATAACT
			Sequence from downstream of group II *Alu* to right HA arm excluding *Alu* sequences	pTO504	AGTTATCCCTCTATCTGTAGG	CTTGCATGCAGTCGACGGGGATCCAAGTTCTGCATCCCCAGGAG

(Continued)

pTO607	UKiS donor (puromycin resistance gene) for *APP* locus	HR110PA-1	*APP* left arm	HCT116 genome	GTAAAACGACGGCCAGTGAATTCAGGCACCCTTGTCAGCGCAA	GAGGTACCGAGCTCGAATTCTAAAGGACTGCTGCTAGGGT
			UKiS marker sequence (puromycin resistance gene), from left insulator to T2A	pTO455	GAATTCGAGCTCGGTACCTC	AGGGCCGGGATTCTCCTCCAC
			UKiS marker sequence (puromycin resistance gene), from T2A to right insulator		GAGGGCAGAGGAAGTCTTCT	CCCTTACGCGATCGCGTTGC
			APP right arm	HCT116 genome	GCAACGCGATCGCGTAAGGGTTTATAGAATAATGTGGGAAGAAACAAAC	CTTGCATGCAGTCGACGGGGATCCCCTTCAAAGAAAAGTCTTGCC
pTO608	UKiS donor (blasticidin resistance gene) for *APP* locus	HR110PA-1	*APP* left arm	HCT116 genome	GTAAAACGACGGCCAGTGAATTCAGGCACCCTTGTCAGCGCAA	GAGGTACCGAGCTCGAATTCTAAAGGACTGCTGCTAGGGT
			UKiS marker sequence (blasticidin resistance gene), from left insulator to T2A	pTO456	GAATTCGAGCTCGGTACCTC	AGGGCCGGGATTCTCCTCCAC
			UKiS marker sequence (blasticidin resistance gene), from T2A to right insulator		GAGGGCAGAGGAAGTCTTCT	CCCTTACGCGATCGCGTTGC
			APP right arm	HCT116 genome	GCAACGCGATCGCGTAAGGGTTTATAGAATAATGTGGGAAGAAACAAAC	CTTGCATGCAGTCGACGGGGATCCCCTTCAAAGAAAAGTCTTGCC
pTO619	UKiS donor (puromycin resistance gene) for *CD44* locus	HR110PA-1	*CD44* left arm	HCT116 genome	GTAAAACGACGGCCAGTGAATTCCCAGAAGGACATAAGGAAAG	GAGGTACCGAGCTCGAATTCACCGAACCTGGCAGAGGCTG
			UKiS marker sequence (puromycin resistance gene), from left insulator to T2A	pTO455	GAATTCGAGCTCGGTACCTC	AGGGCCGGGATTCTCCTCCAC
			UKiS marker sequence (puromycin resistance gene), from T2A to right insulator		GAGGGCAGAGGAAGTCTTCT	CCCTTACGCGATCGCGTTGC
			CD44 right arm	HCT116 genome	GCAACGCGATCGCGTAAGGGGAGAGGCCAGCAAGTCTCAG	CTTGCATGCAGTCGACGGGGATCCGGACCCAGACAGTGCTGGTT
pTO620	UKiS donor (blasticidin resistance gene) for *CD44* locus	HR110PA-1	*CD44* left arm	HCT116 genome	GTAAAACGACGGCCAGTGAATTCCCAGAAGGACATAAGGAAAG	GAGGTACCGAGCTCGAATTCACCGAACCTGGCAGAGGCTG
			UKiS marker sequence (blasticidin resistance gene), from left insulator to T2A	pTO456	GAATTCGAGCTCGGTACCTC	AGGGCCGGGATTCTCCTCCAC
			UKiS marker sequence (blasticidin resistance gene), from T2A to right insulator		GAGGGCAGAGGAAGTCTTCT	CCCTTACGCGATCGCGTTGC
			CD44 right arm	HCT116 genome	GCAACGCGATCGCGTAAGGGGAGAGGCCAGCAAGTCTCAG	CTTGCATGCAGTCGACGGGGATCCGGACCCAGACAGTGCTGGTT
pTO621	UKiS donor (puromycin resistance gene) for *MET* locus	HR110PA-1	*MET* left arm	HCT116 genome	GTAAAACGACGGCCAGTGAATTCGAGAGCCGGAACGAACTCAA	GAGGTACCGAGCTCGAATTCTCCGCGGGTTCCGAGGACCG
			UKiS marker sequence (puromycin resistance gene), from left insulator to T2A	pTO455	GAATTCGAGCTCGGTACCTC	AGGGCCGGGATTCTCCTCCAC
			UKiS marker sequence (puromycin resistance gene), from T2A to right insulator		GAGGGCAGAGGAAGTCTTCT	CCCTTACGCGATCGCGTTGC
			MET right arm	HCT116 genome	GCAACGCGATCGCGTAAGGGTCGCAAGCAATTGGAAACAA	CTTGCATGCAGTCGACGGGGATCCCCACAATCTGTCCAGACACAT
pTO622	UKiS donor (blasticidin resistance gene) for *MET* locus	HR110PA-1	*MET* left arm	HCT116 genome	GTAAAACGACGGCCAGTGAATTCGAGAGCCGGAACGAACTCAA	GAGGTACCGAGCTCGAATTCTCCGCGGGTTCCGAGGACCG
			UKiS marker sequence (blasticidin resistance gene), from left insulator to T2A	pTO456	GAATTCGAGCTCGGTACCTC	AGGGCCGGGATTCTCCTCCAC
			UKiS marker sequence (blasticidin resistance gene), from T2A to right insulator		GAGGGCAGAGGAAGTCTTCT	CCCTTACGCGATCGCGTTGC
			MET right arm	HCT116 genome	GCAACGCGATCGCGTAAGGGTCGCAAGCAATTGGAAACAA	CTTGCATGCAGTCGACGGGGATCCCCACAATCTGTCCAGACACAT
pTO625	Mutating payload for creating *APP* all intron deleted cell	HR110PA-1	sequence from *APP* left arm to 1st exon	HCT116 genome	GTAAAACGACGGCCAGTGAATTCAGGCACCCTTGTCAGCGCAA	TGCTGTGCGAGTGGGATC
			sequence from *APP* 1st exon to right arm	HCT116 cDNA	GATCCCACTCGCACAGCA	CTTGCATGCAGTCGACGGGGATCCCCTTCAAAGAAAAGTCTTGCC

(Continued)

pTO631	Mutating payload for creating *CD44* all intron deleted cell	HR110PA-1	sequence from *CD44* left arm to 1st exon	HCT116 genome	GTAAAACGACGGCCAGTGAATTCCCAGAAGGACATAAGGAAAG	GAGGACGGGACGAGGATG
			sequence from *CD44* 1st exon to right arm	HCT116 cDNA	CATCCTCGTCCCGTCCTC	CTTGCATGCAGTCGACGGGGATCCGGACCCAGACAGTGCTGGTT
pTO632	Mutating payload for creating *MET* all intron deleted cell	HR110PA-1	sequence from *MET* left arm to 1st exon	HCT116 genome	GTAAAACGACGGCCAGTGAATTCGAGAGCCGGAACGAACTCAA	CTCCTCTCAGCAAGTCAGCT
			sequence from *MET* 1st exon to right arm	HCT116 cDNA	AGCTGACTTGCTGAGAGGAG	CTTGCATGCAGTCGACGGGGATCCCCACAATCTGTCCAGACACAT
pTO454	*TP53*-specific gRNA/Cas9 expression plasmid for UKiS 1st step in HCT116 or iPS cells	pX330	gRNA-TP53 (R) target sequence	Oligonucleotide only	CACCGCTCAGAGGGGGCTCGACGCT	AAACAGCGTCGAGCCCCCTCTGAGC
pTO639	*TP53*-specific gRNA/Cas9 expression plasmid for UKiS 1st step in iPS cells	pX330	gRNA-TP53 (L) target sequence	Oligonucleotide only	CACCGGTGCTTTAAGAATTACCGC	AAACGCGGTAATTCTTAAAGCACC
pTO546	*APP*-specific gRNA/Cas9 expression plasmid for UKiS 1st step in HCT116 cells	pX330	gRNA-APP (L) target sequence	Oligonucleotide only	CACCGCTCCCGGGGGTGTCGTATAA	AAACTTATACGACACCCCCGGGAGC
pTO548	*APP*-specific gRNA/Cas9 expression plasmid for UKiS 1st step in HCT116 cells	pX330	gRNA-APP (R) target sequence	Oligonucleotide only	CACCGTTCTATAAATGGACACCGAT	AAACATCGGTGTCCATTTATAGAAC
pTO595	*CD44*-specific gRNA/Cas9 expression plasmid for UKiS 1st step in HCT116 cells	pX330	gRNA-CD44 (L) target sequence	Oligonucleotide only	CACCGCGAGGATGGCGGACCGAACC	AAACGGTTCGGTCCGCCATCCTCGC
pTO599	*CD44*-specific gRNA/Cas9 expression plasmid for UKiS 1st step in HCT116 cells	pX330	gRNA-CD44 (R) target sequence	Oligonucleotide only	CACCGCCAAGTGGACTCAACGGAG	AAACCTCCGTTGAGTCCACTTGGC
pTO603	*MET*-specific gRNA/Cas9 expression plasmid for UKiS 1st step in HCT116 cells	pX330	gRNA-MET (L) target sequence	Oligonucleotide only	CACCGGGCCGCGCGCGCCGATGCC	AAACGGCATCGGCGCGCGCGGCCC
pTO604	*MET*-specific gRNA/Cas9 expression plasmid for UKiS 1st step in HCT116 cells	pX330	gRNA-MET (R) target sequence	Oligonucleotide only	CACCGTTCCCACCTCGCAAGCAAT	AAACATTGCTTGCGAGGTGGGAAC
pTS195	Off-Target Less gRNA/Cas9 expression plasmid for UKiS 2nd step	pX330	TL-gRNA target sequence	Oligonucleotide only	CACCGGCGCAACGCGATCGCGTAA	AAACTTACGCGATCGCGTTGCGCC

Primers used for plasmid sequencing

Target	Sequence
UKiS 1st step donor plasmids	CTGCAAGGCGATTAAGTTGG
	GGGCTGTCCCTCATAAAAGT
	ACCCGTTGCGAAAAAGAAC
	ACAGTCCCCGAGAAGTTGG
	ATGGGTACCGTGAGCAAGG
	AGCAGAAGAACGGCATCAAGGT
	GAGGGCAGAGGAAGTCTTCT
	CAGACATGATAAGATACATTGATG
	AACTTGTTTATTGCAGCTTA
	TCACGTAAGTAGAACATGAAATAACC
	CCCTTACGCGATCGCGTTGC
	TATGCTTCCGGCTCGTATGT
UKiS 2nd step donor plasmids for *TP53* 1st intron mutation	CTGCAAGGCGATTAAGTTGG
	CCTGGTCTGAAGGACAGCA
	GGTAATTCTTAAAGCACCTGCAC
	CTCCCCAACTCCATTTCCTT
	TCTCGGCTCCGTGTATTTTC
	CCCATGGCATCCTAGTGAAA
	AATCCCTCCCCTTCTTTGAA
	TCCTAAATCGAGGTGGCATT
	GGAGAACTTCATATAGAATGGAATCA
	GGAATTGCGAGTTTGGAAGT
	TTAACCCCAGGGTCATGAAG
	TCTTAGTGCTATTTTCTATTTATCTCC
	CCAATTTTAGCAAACCAAACC
	ACATTCTAACCAAATAATGTTTA
	CATAATGGTAGAAAACTCAGTGCT
	CGCTTTTTCCTCTAAGATCAGG
	CCCATTAGGACATGTATGTATAGAA
	AATAACGATTAGAAAGGAAGAAATA
	AATAACGATTAGAAAGGAAGAAATA
	CCCATTAGGACATGTATGTATAGAA
	AGTGAAACTCTATCTCAAAAA
	AGGGCACCTGCATTTCTCTT
	TACTGTGTGCCCAGCACTTT
	GCTGTTTGGTAGGGGATGTT
	GTGTAACAAACACTTACAGTAG
	AAACATGGCAAAGCCTTTGA
	GAATAACACACAAGCCTGTTATA
	AGGTGAGCGGTGAGACAGTT
	ACAGGAAGGGAGTTGGGAATA
	CTTCCTGAAAACAACGTTCTG
	TATGCTTCCGGCTCGTATGT

(Continued)

UKiS 2nd step donor plasmid for *APP* all introns deletion	CTGCAAGGCGATTAAGTTGG
	AGCGGTAGGCGAGAGCAC
	AACCCCAGATTGCCATGTT
	CCACTGGCTGAAGAAAGTGAC
	TTGCCCGAGATCCTGTTAAA
	TGACACACCTCCGTGTGATT
	TGCAGAAGATGTGGGTTCAA
	TGCATGAATAGATTCTCTCCTGA
	TGCTGGTCTTCAATTACCAAGA
	TATGCTTCCGGCTCGTATGT
UKiS 2nd step donor plasmid for *CD44* all introns deletion	CTGCAAGGCGATTAAGTTGG
	CCAGCCTCTGCCAGGTTC
	CCACGTGGAGAAAAATGGTC
	CCATCCCAGACGAAGACAGT
	CCATGAGCATCATGAGGAAG
	GGAATGATGTCACAGGTGGA
	GAGAGGCCAGCAAGTCTCAG
	CAGGGTTAATAGGGCCTGGT
	TATGCTTCCGGCTCGTATGT
UKiS 2nd step donor plasmid for *MET* all introns deletion	CTGCAAGGCGATTAAGTTGG
	GAGCGCCTCAGTCTGGTC
	CGCCGTGATGAATATCGAAC
	GAGGGACAAGGCTGACCATA
	TTTATTAGTGGTGGGAGCACAA
	TTCAACCGTCCTTGGAAAAG
	AAGAGGGCATTTTGGTTGTG
	AGTGAAGTGGATGGCTTTGG
	GGCCATCGATATTCTTTGCT
	GGCTGTAGTGCAGTGGTGTG
	CAGCATGTTTGTAAAGCAGGA
	GAAAAGGTATGTCAGACTGGGATT
	TATGCTTCCGGCTCGTATGT
gRNA/Cas9 expression plasmids	GGAAAGTCCCTATTGGCGTTA

Supplementary Table 5. Primers used for off-target analysis.

Primers used for PCR

Fig. #	Target	Forward primer	Reverse primer
Supplementary Fig. 2	gRNA-TP53 (R) off-target candidate #1	ACAGAGGCCCCCAAATAAG	TGACTGTGTCTGGAGGATGG
	gRNA-TP53 (R) off-target candidate #2	GAACCTCCAGTGTGTGAAAGG	ATACAAGAAATGCCCCCAAA
Supplementary Fig. 3	TL-gRNA off-target candidate	CGACCTTCTGGGAGCAGT	TCCACAGGTACGACAACGAA

Primers used for sequencing

Fig. #	Target	primer
Supplementary Fig. 2	gRNA-TP53 (R) off-target candidate #1	ACAGAGGCCCCCAAATAAG
	gRNA-TP53 (R) off-target candidate #2	GAACCTCCAGTGTGTGAAAGG
Supplementary Fig. 3	TL-gRNA off-target candidate	CGACCTTCTGGGAGCAGT

Supplementary Table 6. Primers used for real-time RT-PCR.

Fig. #	Target	Forward primer	Reverse primer
Fig. 5c, 6b-c and Supplementary Fig. 8	*TP53* mRNA	GACTGCCTTCCGGGTCACT	GGACAGCATCAAATCATCCA
	18S rRNA	ATTAATCAAGAACGAAAGTCGGAGGT	TTTAAGTTTCAGCTTTGCAACCATACT
	CDKN1A mRNA	AGGGGACAGCAGAGGAAGAC	GGCGTTTGGAGTGGTAGAAA
	GADD45A mRNA	GGCCCGGAGATAGATGACTT	GTTTTCCTTCCTGCATGGTT
	BAX mRNA	CATGTTTTCTGACGGCAACT	CCGGAGGAAGTCCAATGTC
	PUMA mRNA	TCAGGAAAGGCTGTTGTGCT	ACGTTTGGCTCATTTGCTCT
	RRM2B mRNA	GCCAGGACTCACTTTTTCCA	CAATGATCTCCCTGACCCTTT
