# Supplementary material for: Biallelic and gene-wide genomic substitution for endogenous intron and retroelement mutagenesis in human cells
Source: Nat Commun. 2022 Jul 21;13:4219. doi: 10.1038/s41467-022-31982-1 (PMC9304424; doi:10.1038/s41467-022-31982-1)
Supplement: Supplementary file 2 — Reporting Summary [file 41467_2022_31982_MOESM2_ESM.pdf]

nature portfolio

Corresponding author(s): Yasunori Aizawa

Last updated by author(s): Jul 10, 2022

Reporting Summary

Nature Portfolio wishes to improve the reproducibility of the work that we publish. This form provides structure for consistency and transparency in reporting. For further information on Nature Portfolio policies, see our Editorial Policies and the Editorial Policy Checklist.

Statistics

For all statistical analyses, confirm that the following items are present in the figure legend, table legend, main text, or Methods section.

n/a	Confirmed	
☐	☒	The exact sample size (*n*) for each experimental group/condition, given as a discrete number and unit of measurement
☐	☒	A statement on whether measurements were taken from distinct samples or whether the same sample was measured repeatedly
☐	☒	The statistical test(s) used AND whether they are one- or two-sided<br>*Only common tests should be described solely by name; describe more complex techniques in the Methods section.*
☒	☐	A description of all covariates tested
☒	☐	A description of any assumptions or corrections, such as tests of normality and adjustment for multiple comparisons
☐	☒	A full description of the statistical parameters including central tendency (e.g. means) or other basic estimates (e.g. regression coefficient) AND variation (e.g. standard deviation) or associated estimates of uncertainty (e.g. confidence intervals)
☐	☒	For null hypothesis testing, the test statistic (e.g. *F*, *t*, *r*) with confidence intervals, effect sizes, degrees of freedom and *P* value noted<br>*Give P values as exact values whenever suitable.*
☒	☐	For Bayesian analysis, information on the choice of priors and Markov chain Monte Carlo settings
☒	☐	For hierarchical and complex designs, identification of the appropriate level for tests and full reporting of outcomes
☒	☐	Estimates of effect sizes (e.g. Cohen's *d*, Pearson's *r*), indicating how they were calculated

Our web collection on statistics for biologists contains articles on many of the points above.

Software and code

Policy information about availability of computer code

Data collection	Flow cytometry data collection: SH800 Cell Sorter software (ver. 2.1.5) or BD FACSChorus software (ver. 1.3)<br>Immunoblot image acquisition: Image Quant LAS 4000mini software (ver. 1.2)<br>Real-time RT-PCR data collection: Thermal Cycler Dice Real Time System software (ver. 6.01A)<br>Confocal microscopy image acquisition: Zen Software Black Edition (ver. 14.0.20.201)
Data analysis	Flow cytometry data analysis: SH800 Cell Sorter software (ver. 2.1.5) or BD FACSChorus software (ver. 1.3)<br>Band density analysis: ImageJ software (ver. 1.51j8)<br>Real-time RT-PCR data analysis: Thermal Cycler Dice Real Time System software (ver. 6.01A)<br>Confocal microscopy image analysis: Zen Software Black Edition (ver. 14.0.20.201)

For manuscripts utilizing custom algorithms or software that are central to the research but not yet described in published literature, software must be made available to editors and reviewers. We strongly encourage code deposition in a community repository (e.g. GitHub). See the Nature Portfolio guidelines for submitting code & software for further information.

Data

Policy information about availability of data

All manuscripts must include a data availability statement. This statement should provide the following information, where applicable:

- Accession codes, unique identifiers, or web links for publicly available datasets
- A description of any restrictions on data availability
- For clinical datasets or third party data, please ensure that the statement adheres to our policy

The data supporting the findings of this study are available within the main body of this manuscript and its Supplementary Information files. The source data

underlying Figs. 2c, 3b-e, 5a,c-e, 6b,c, and 7a,c-h, as well as Supplementary Figs. 2b, 3c, 4c, 6, 8, 9, 11b, 13b, 14b, and 15b are provided as a Source Data file. The genomic positions of the exon/intron boundaries and of retroelements were referred from the tracks of NCBI RefSeq genes and RepeatMasker, respectively, in the USCS Genome Browser (https://genome.ucsc.edu/index.html). All SNPs referred in this article are registered in dbSNP151 (https://www.ncbi.nlm.nih.gov/projects/SNP/snp_summary.cgi).

Field-specific reporting

Please select the one below that is the best fit for your research. If you are not sure, read the appropriate sections before making your selection.

- [x] Life sciences
- [ ] Behavioural & social sciences
- [ ] Ecological, evolutionary & environmental sciences

For a reference copy of the document with all sections, see nature.com/documents/nr-reporting-summary-flat.pdf

Life sciences study design

All studies must disclose on these points even when the disclosure is negative.

Sample size	- Sample size for evaluation on efficiencies of our genome modification: we decided to collect 5-10 cell clones after the drug selection and FACS sorting at the 1st and 2nd steps of UKiS, respectively. This sample size was determined in our preliminary experiment in order to obtain at least 3 clones had the expected genome modification.<br>- Sample size for cell clone characterization (Junction PCR, agarose gel electrophoresis, RT-PCR, real-time RT-PCR, immunoblotting & immunochemical staining): We decided that 3 cell clones were examined as biological triplicates for every mutant.
Data exclusions	No data was excluded.
Replication	- Junction PCR, agarose gel electrophoresis, RT-PCR, real-time RT-PCR, immunoblotting & immunochemical staining: 3 biological replicates and 3 technical replicates for every biological replicate.<br>- SNP typing & off-target analyses: 3 biological replicates and one technical replication for every biological replicate.
Randomization	Randomization for cell clone characterization: since the 3 clones of every mutant were randomly chosen out of the clones obtained at the 2nd step of UKiS, no bias should be present to evaluate the effects of genome modification that we introduced.
Blinding	Most of the experiments were performed by different co-authors. The data were also processed and re-evaluated by more than two authors.

Reporting for specific materials, systems and methods

We require information from authors about some types of materials, experimental systems and methods used in many studies. Here, indicate whether each material, system or method listed is relevant to your study. If you are not sure if a list item applies to your research, read the appropriate section before selecting a response.

Materials & experimental systems

n/a	Involved in the study	
[ ]	[x]	Antibodies
[ ]	[x]	Eukaryotic cell lines
[x]	[ ]	Palaeontology and archaeology
[x]	[ ]	Animals and other organisms
[x]	[ ]	Human research participants
[x]	[ ]	Clinical data
[x]	[ ]	Dual use research of concern

Methods

n/a	Involved in the study	
[x]	[ ]	ChIP-seq
[ ]	[x]	Flow cytometry
[x]	[ ]	MRI-based neuroimaging

Antibodies

Antibodies used	Mouse monoclonal anti-p53, clone DO-1 (Santa Cruz Biotechnology, sc-126, 1:2,000)<br>Mouse monoclonal anti-α-Tubulin, clone 10G10 (Wako, 017-25031, 1:5,000)<br>Mouse monoclonal anti-NANOG, clone 1E6C4 (Santa Cruz Biotechnology, sc-293121, 1:500)<br>Rabbit polyclonal anti-OCT4 (Abcam, ab19857, 1:500)<br>Anti-mouse IgG, HRP-Linked Whole Ab Sheep (GE Healthcare, NA931-1ML, 1:5,000)<br>Alexa Fluor 488-conjugated goat anti-rabbit IgG (Invitrogen, A-11008, 1:1,000)<br>Alexa Fluor 568-conjugated goat anti-mouse IgG (Invitrogen, A-11004, 1:1,000)
Validation	Mouse monoclonal anti-p53 , clone DO-1 (Santa Cruz Biotechnology, sc-126) has been previously validated by the manufacturer (more information is available at https://www.scbt.com/p/p53-antibody-do-1?productCanUrl=p53-antibody-do-1&_requestid=1813467) and cited at least 5974 times.<br>Mouse monoclonal anti-α-Tubulin, clone 10G10 (Wako, 017-25031) has been previously validated by the manufacturer (more information is available at https://labchem-wako.fujifilm.com/us/product/detail/W01W0101-2503.html) and cited at least 13 times.

Mouse monoclonal anti-NANOG, clone 1E6C4 (Santa Cruz Biotechnology, sc-293121) has been previously validated by the manufacturer (more information is available at https://www.scbt.com/p/nanog-antibody-1e6c4?productCanUrl=nanog-antibody-1e6c4&_requestid=1814415) and cited at least 105 times.
Rabbit polyclonal anti-OCT4 (Abcam, ab19857) has been previously validated by the manufacturer (more information is available at https://www.abcam.com/oct4-antibody-ab19857.html) and cited at least 458 times.

Eukaryotic cell lines

Policy information about cell lines

Cell line source(s)	HCT116 cells, American Type Culture Collection, CCL-247<br>201B7 iPS cells, RIKEN BRC, HPS0063
Authentication	Both HCT116 cells and 201B7 iPS cells were authenticated by their respective suppliers using STR profiling.
Mycoplasma contamination	Not tested.
Commonly misidentified lines (See ICLAC register)	No commonly misidentified cell lines were used in this study.

Flow Cytometry

Plots

Confirm that:

- [x] The axis labels state the marker and fluorochrome used (e.g. CD4-FITC).
- [x] The axis scales are clearly visible. Include numbers along axes only for bottom left plot of group (a 'group' is an analysis of identical markers).
- [ ] All plots are contour plots with outliers or pseudocolor plots.
- [ ] A numerical value for number of cells or percentage (with statistics) is provided.

Methodology

Sample preparation	Approximately 14 hours after transfection, cells were washed with DPBS, trypsinized for 3 min at 37°C, and suspended in culture media, centrifuged gently (150 g for 3 min) to remove supernatant. And then, cells were resuspended in 500 µL of culture media, filtered through the 40 µm cell strainer and transferred to tubes for flow cytometry analysis/sorting.
Instrument	SH800 Cell Sorter (Sony Biotechnology) or BD FACSMelody Cell Sorter (Becton, Dickinson and Company)
Software	Data were collected and analyzed using SH800 Cell Sorter software (ver. 2.1.5) or BD FACSChorus software (ver. 1.3).
Cell population abundance	The populations of GFP negative cells and positive cells are given in detail in Figure 3 and Supplementary Figure 6, 9, 11c, 13c, 14c and 15c. We confirmed by fluorescence microscopy that most of sorted cells were GFP negative as expected.
Gating strategy	For TP53 mutagenesis in HCT116 cells, FSC-A vs BSC-A was used to exclude cell debris. For TP53 mutagenesis in iPS cells and CD44, MET and APP mutagenesis in HCT116 cells, FSC-A vs SSC-A was used to exclude cell debris, and then two more filtering steps (SSC-H vs SSC-W and FSC-H vs FSC-W) were used to select single isolated cells. The gating strategies are depicted in details in Supplementary figures 19-21.

- [x] Tick this box to confirm that a figure exemplifying the gating strategy is provided in the Supplementary Information.
